# Analysis of electric vehicle charging station usage and profitability in Germany based on empirical data

**DOI:** 10.1016/j.isci.2022.105634

**Published:** 2022-11-19

**Authors:** Christopher Hecht, Jan Figgener, Dirk Uwe Sauer

**Affiliations:** 1 Institute for Power Electronics and Electrical Drives, RWTH Aachen University, Aachen, Germany; 2 Institute for Power Generation and Storage Systems, E.ON ERC, RWTH Aachen University, Aachen, Germany; 3 Juelich Aachen Research Alliance, JARA-Energy, Aachen, Germany; 4 Helmholtz Institute Muenster (HI MS), IEK-12, Forschungszentrum Jülich, Jülich, Germany

**Keywords:** Energy management, Energy Modelling, Energy policy, Energy resources

## Abstract

This paper provides empirical data and a profitability estimation of public charging infrastructure usage in Germany. Given that, in Germany, there are now 2.5 times as many vehicles per charging station compared with 2017, the system needs to allocate charging points efficiently. To this end, this paper presents representative data on energy consumption, arrival times, occupation, and estimated profitability of 22,200 charging stations in Germany. The observed patterns are translated into compact empirical models that allow working with the results without the burden of the large-scale datasets. Charging happens mainly during the day and on weekdays for AC charging stations, whereas DC fast-charging stations are more popular on the weekend. Fast-chargers service approximately three times as many vehicles per charge point at higher profits because of better margins. For AC chargers, up to 20 kWh of energy are charged in an average charge event, whereas fast-chargers supply approximately 40 kWh. Energy transfer typically terminates after 4 h for AC chargers and 45 min for fast-chargers. The power rates are significantly below the rated station power and rarely exceed 11 kW for AC charging. This paper allows fellow researchers to build simulation and test scenarios using presented data or to verify models.

## Highlights

• Usage analysis of 22,000 German public charging stations
• Profitability analysis by the area type and power level
• Discussion of usage patterns by the area type, power level, and connector type
• Highly inhomogeneous usage patterns across area types and power levels analyzed

## Graphical abstract

**Figure:**
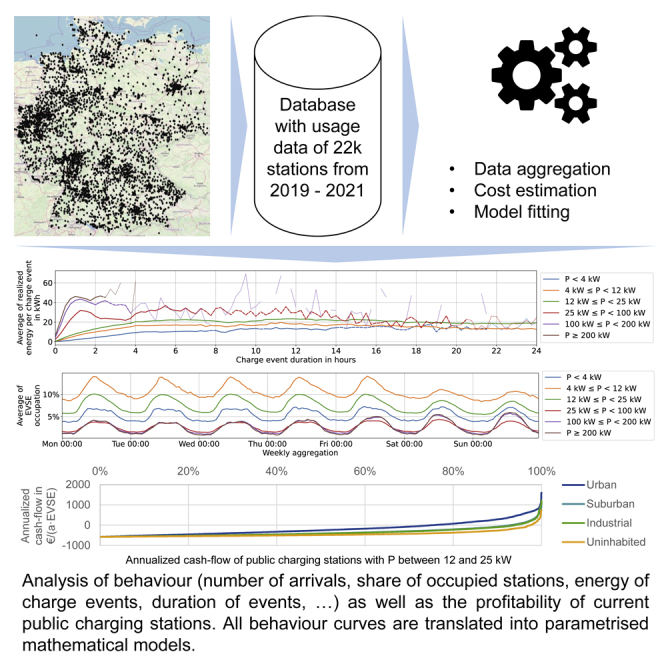

## Introduction

Climate change is a key challenge of the 21st century. To tackle this issue, greenhouse gas emissions must be reduced across all sectors of human activity. Whereas progress has been made in many sectors, the traffic sector is particularly hard to decarbonize; emissions have risen by 74% between 1990 and 2016 worldwide.^1^ One approach to do so is to electrify transport. For on-road electric vehicles (EVs), this requires public recharging opportunities (see Figure 2 for a component overview). Such public charging stations (PCS) are expensive to install. To make such investments worthwhile, sufficient knowledge of PCS usage is required. Two typical ways to generate this knowledge are to analyze field data and to model user behavior or traffic. Building on a large dataset, this paper follows both approaches. We first present the results of a large-scale data analysis based on the PCS usage in Germany between 2019 and 2021. The involved amount of data is hundreds of gigabytes, which makes working with the full dataset computationally challenging. For this reason, this paper also defines empirical models in the supplemental information, which capture both the mean values as well as uncertainties of relevant quantities. These models are defined by 6 or 12 fitted parameters that capture the relevant behavior. They can therefore be integrated into code running in low-memory environments.

The observed patterns shall thereby inform PCS operators, navigation system providers, grid operators, energy system modelers, and many more. PCS operators may use this knowledge to optimize pricing strategies and infrastructure construction. Navigation system providers require this information to ensure that drivers find an unoccupied recharging opportunity either on the way or upon arriving at their destination. Grid operators need to dimension electricity grids supplying PCSs to a realistic demand. Energy system modelers need to understand how much energy is required when and where for power generation and storage to meet demand.

**Figure 1:**
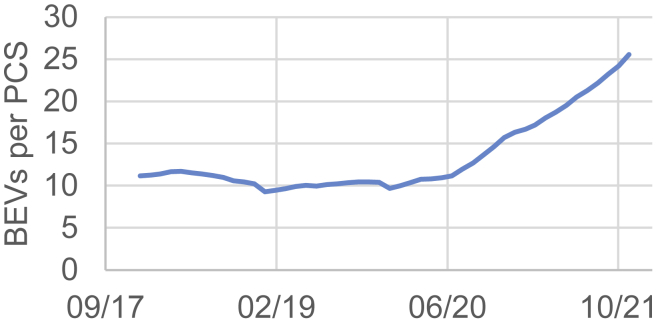
BEVs per PCS in Germany^2^^,^^48^

These questions gain an increasing urgency worldwide. Taking Germany as an example, the number of battery electric vehicles (BEVs) that have to share a single PCS is steadily increasing (see Figure 1) reaching approximately 25 BEVs per PCS by the end of 2021. To avoid excessive queuing at PCS, all actors mentioned need to increase the intelligence of their system.

In this paper, we provide a detailed analysis of how, where, and when PCSs are used. The underlying research goal is to understand and characterize PCS usage as well as to determine their profitability. This is achieved in a two-step process using a dataset comprised of 27,800 PCSs in Germany observed in the three years from 2019 to 2021. After filtering non-usable data, the number of usable PCS is reduced to 22,200 PCS. The public registry of the German government listed 25,900 PCSs by the end of 2021.^2^ The difference may be a result of delays with the reporting to the government website or unfound duplicates in our dataset. Unfortunately, naming conventions are not unified between the two datasets. One way of verifying the overlap is to check how close stations are to each other. For 75.7% of PCS in the government’s list, we find a PCS in our filtered dataset within 300 m and, similarly, we find a PCS in the government’s list within 300 m for 83.9% of PCS in our dataset. From this finding, we can conclude that there is a significant overlap between the two datasets and that trends can be assumed to be representative.

The study is unmatched in terms of amount and representativeness.^3^^,^^4^^,^^5^^,^^6^^,^^7^^,^^8^^,^^9^^,^^10^^,^^11^ In the first block of the result section, the dataset is visualized to answer the following research question:

I – When and for how long are PCSs typically occupied and how much energy is consumed per charge event?

Next, the data are analyzed in terms of station profitability:

II – How profitable are PCSs?

In the supplemental information, the data are used for the mathematical analysis of charging infrastructure usage. The key question to be answered in this regard is:

III – How can empirical models be fitted that incorporate the answers to question I?

Collectively, answering these three questions achieves the stated research goal. To contextualize these questions, the current state of PCS deployment in Germany is presented alongside selected information on how users typically interact with PCSs in the following.

### Literature review

This section starts by providing naming conventions and fundamentals of how PCSs are currently used and paid for. The second half then introduces the current state of scientific literature.

**Figure 2:**
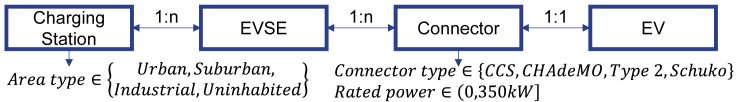
Relationship between the components and properties of a (public) charging station in the context of this work

Each PCS contains one or many electric vehicle supply equipment units (EVSEs) that are able to serve a vehicle. One EVSE may have one or many connectors installed. Typical AC chargers have only a single Type 2 or Schuko connector installed, whereas DC chargers often have a CCS and a CHAdeMO connector installed. If multiple connectors are installed, only one of them can be used at any moment in time. An overview of this chain of relationships can be found in Figure 2.

**Figure 3:**
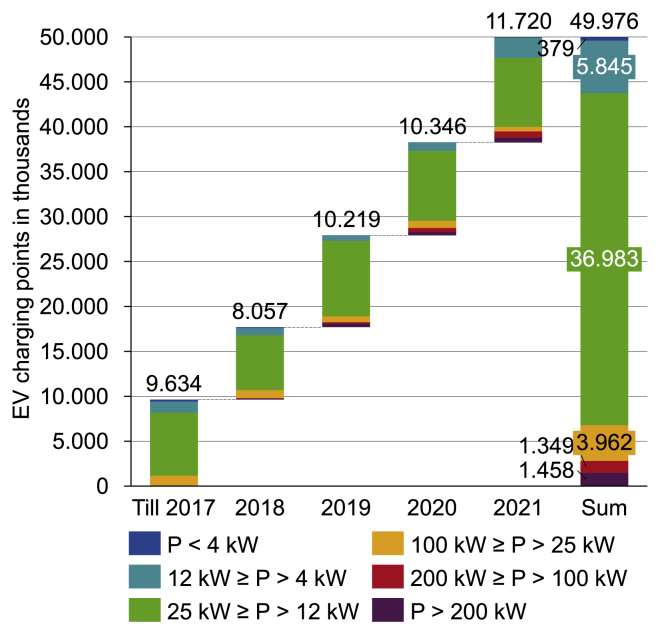
Installed charge points by power level in Germany by year of installation. Data taken from Figgener et al.,^12^ which are based on Bundesnetzagentur^2^

Public charging infrastructure in Germany has grown in a near-linear fashion over the last years with between 8,000 and 11,700 charge points added annually^12^ as Figure 3 shows. 75% of these charge points were installed at PCS rated between 12 and 25 kW with virtually all of these installations providing two charge points per PCS. For faster PCS with a rated power ≥100 kW, the picture is less clear. By the end of 2021, 650 PCSs consisted of only a single charge point, 1,230 had 2, 313 had 3, and 53 had 4 or more charge points.^2^ The latter category is also the most relevant one for highway traveling, as these typically are located at refueling stations on highways. To increase coverage, the government is planning a grid of 1,000 fast-chargers in the so-called “DeutschlandNetz.”^13^

**Figure 4:**
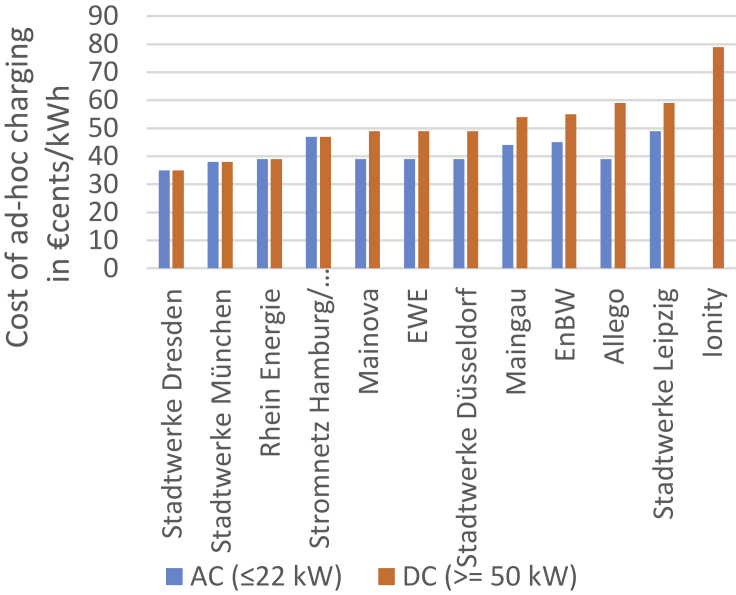
Comparison of prices for ad-hoc charging at PCS by charge point operator^61^

As no exhaustive dataset exists on how users pay for charging services, surveys are typically used instead.^14^^,^^15^^,^^16^ According to Shell Recharge Solutions,^14^ 36% of users have four or more charging cards that they use to authenticate themselves at PCS. These charging cards are connected to accounts at e-mobility service providers who usually bill customers on a monthly basis. The high number of cards and accounts is necessary as interoperability is not always guaranteed despite the fact that users would be willing to pay for the premium service of having to use only a single card or other types of payment.^14^ Given that charge point operator and e-mobility service providers do not have to be the same entity, prices are very diverse and frequently not transparent. Figure 4 compares charging prices based on spontaneous ad-hoc charging without a contract at each operator. As can be seen, prices were between 30 and 50 €cents/kWh for most operators at the time of data collection. In terms of overall energy transferred, fast-chargers supply 23% of energy charged by users, whereas slower AC chargers supply only 16%.^16^ The remaining energy is recharged at home (45%) or at the workplace (16%).

Having discussed reports and statistics on current PCS usage, we now introduce concepts and approaches that scholars have previously used. In the early days of PCS deployment, little empirical data were available.^17^^,^^18^^,^^19^^,^^20^ Scholars, therefore, had to use traffic counter data,^21^^,^^22^ socio-economic models, and expert opinions.^23^^,^^24^ When using traffic counter data, it was assumed that a certain share of passing vehicles was electric and required recharging. Socio-economic models estimate correlations between EV recharging needs and datasets describing social or economic parameters of certain areas. Lastly, expert opinions are similar to socio-economic models. However, they use expert knowledge to develop models with much less required data input. The challenge with the three options is that although large datasets are available, the link between the data and the actual PCS usage is hard to prove.

When using EV field data, a typical approach is to assume that vehicles charge where they are parked.^25^^,^^26^ This allows for the derivation of a model, which would indicate where PCSs would be most occupied. The issue with this approach is that monitored vehicle fleets are typically quite small and representativeness is not a safe assumption.

With the increasing market share of EVs in many countries, researchers took a new approach: large datasets from PCS usage started to become available. Works were performed around Amsterdam and its surroundings,^6^^,^^7^ the Netherlands as a whole,^8^^,^^9^^,^^10^ Nebraska, USA,^3^^,^^4^^,^^5^ and Jiangsu Province, China.^11^ The level of detail differs between each study. Generally, the occupation status per PCS is known. To a lesser extent, the energy per charge even was also known. Note that the authors of the studies are overlapping indicating that fewer datasets are available than papers published. An overview of which study contains what kind of information has been provided by Fischer et al.^27^ and Calearo et al.^28^ A key remaining issue is a representativeness, particularly if the area under observation contains only a small population such as Amsterdam or Nebraska.

To tackle the challenge of representativeness, we have previously created a study looking at the German PCSs^29^^,^^30^ where a large share of the PCSs in the country was under observation for the three months before the COVID-19 pandemic. The same data were also used to train regression models to identify attractive sites for new charging stations based on their immediate environment as identified by nearby OpenStreetMaps points of interest.^31^ As the data used in the previous study were obtained from publicly accessible websites, no information about the amount of energy charged, or the users were available. Data quality was limited by the collection method.

### This paper in the context of literature

This paper solves the previously existing issues by building on an exhaustive dataset provided by the industry. This allows for a first-of-its-kind study in terms of the data depth, quality, and area covered. The provided data encompasses the years 2019 until 2021 and provides PCS status for several ten thousand PCS. This study further utilizes approximately 9 million charge detail records (CDRs) to provide insights into the energy demand distribution across charging processes. This rich dataset allows us to make statements regarding infrastructure use, user behavior, and PCS profitability – each in a representative manner for Germany; the leading EV market by number of vehicles sold in Europe at the time of writing. Specifically, this study is able to provide estimates on how much energy is consumed per charge event or PCS and how profitable PCSs are. These aspects were not discussed at the given level of detail and scale in recent years. Other aspects such as occupation levels, durations, and arrival rates were discussed, but not with the same data validity. The quickly developing market and the changing properties of EVs mean that even if previous studies exist – such as Wolbertus et al. (2016),^7^ Wolbertus et al. (2018),^8^ Wolbertus et al. (2017),^32^ and Hecht et al.^29^ – these can quickly become outdated and need to be augmented by newer studies such as this one.

### Structure of this document

It is in the nature of data-driven analyses that insights can already be achieved by carefully aggregating and displaying data. For the remainder of the introduction section, we therefore decided to outline the data sources, data pre-processing first, and methodology.

Within the results section, the insights from data aggregation and visualization are shown first. With these visualizations, we share key aspects that modelers should observe when evaluating whether their own models accurately represent real-world behavior. The second part of the results are those that are based on the methodology described. The paper terminates with a discussion of both segments – the data visualizations and the results.

#### Data sources and pre-processing

Given that the data sources used in this paper are crucial for all subsequent results, this section explains the data sources and data pre-processing steps used. For the sake of conciseness, we did not include an extensive discussion on why which metric was chosen in the main body of this paper. This can be found in section IV of the supplementary information.

##### Data sources

This section lists data sources in this document. These consist of the PCS data as the main dataset. This data are split into the more precise charge detail records (CDRs) and the PCS status data. CDRs hold information about individual charge events such as start and end time or energy transferred. PCS status data lists when a PCS is occupied or out of order. The second data source provides area types based on the Corine land cover model of the European Union.^33^ Other external data sources such as weather data, traffic information, or public holidays were also available to the authors, but are not included in this paper as a previous study showed that they had limited explanatory value.^34^ The correlation and share of PCS within the different land use categories, power levels, etc., can be found in section III of the supplementary information.

##### PCS data

In the context of the project BeNutz LaSA,^35^ our industry partners Hubject and SMART/LAB provided us with usage data of 27,800 PCSs. Both datasets contain the location of each PCS, the ids and rated power of the connected EVSEs, the connectors available at each EVSE, and the status changes of the EVSEs. To simplify the analysis and ensure consistency across the dataset, we limit our analysis to the statuses “occupied,” “available,” and “unknown.” Other status reports such as “reserved,” “preparing,” etc., were mapped as one of the three selected, as outlined in.^29^

A second key piece of data are the CDRs provided by the industry partners. Such CDRs were available for all charge events at PCSs operated by SMART/LAB as the company is a backend operator. For the data obtained from Hubject, CDRs were only available for charge events handled by the roaming network of Hubject, which represents a minority of charge events. Each record contains details about a charge event such as the start and end time, the energy that was consumed during the event, and the id of the used EVSE. For one of the partners, the records further contained an anonymized id of the contract and the authentication token used.

Note that neither dataset provides any information about instantaneous power flow. This means that charge events end when the cable is unplugged and not when no more power flows.

##### Area types

The Corine land cover model^33^ was used to identify the area type of PCSs. The model assigns a land usage type to land across the European Union. The available land use categories were grouped into the categories Urban, Suburban, Industrial, Uninhabited, and Non-Fitting (for details, see ^29^).

##### Cost information

In this paper, we estimate the profitability of PCS. To do so, several estimations of costs and revenue are required. Alberer^36^ performed an extensive literature review and finds the cost metrics shown in Table 1. Unfortunately, the author did not provide an indication of how many EVSEs there are per PCS. It is, however, clear that the price assumptions vary widely across literature, which corresponds to the industry experience of the authors. Costs are heavily influenced by land acquisition, selection of hardware suppliers, project management costs, and many other factors.

**Table 1:** Cost estimation for PCS^36^

Power level	Metric	Value
50 kW	Median	815 €/kW
	Average	1,130 €/kW
	Minimum	480 €/kW
	Maximum	2,200 €/kW
150–250 kW	Median	460 €/kW
	Average	504 €/kW
	Minimum	393 €/kW
	Maximum	800 €/kW

The uncertainty is even greater for AC PCSs with a power rating of 11 or 22 kW. Typically, these two are grouped into the same category, as they are very similar in structure. Literature provides investment estimations per PCS with large uncertainty bands such as 6,500–14,000 €,^37^ 7,500–15,000 €,^38^ 7,500 €,^39^ 2,000–5,000 €.^40^ Large influencing factors for these variations are hardware costs for which one study estimates costs between 2,500 € and 8,000 €,^37^ and installation costs, which vary from 500 € to 3,000 €.^41^ Wallboxes with a rated power of 3.7 kW are seen as somewhat cheaper with investment costs of 2,700 €.^39^ Depending on the location, however, installation costs can become a significant factor^37^ for this category as well. Given these findings, we make the following assumptions stated in Table 2 for CapEx in this study.

**Table 2:** Chosen CapEx per power level

Power level	CapEx per PCS	Reasoning
*P* < 4 kW	2,000 €	The cost estimation from literature is slightly older. Economy of scale likely reduced hardware costs.
4 ≤ *P* < 12 kW	6,000 €	Lower end of values stated in literature because of low power level. Slight reduction compared with literature to account for economy of scale.
12 ≤ *P* < 25 kW	9,000 €	Mid-range from literature
25 ≤ *P* < 100 kW	50,000 €	Slightly below average value from literature as some sources were quite old and therefore likely more expensive
100 ≤ *P* < 200 kW	100,000 €	Slightly above values from literature to account for the fact that PCS have more EVSEs than 2, which is assumed in many studies
*P* ≥ 200 kW	200,000 €	

To annualize these investment costs, it is necessary to estimate the lifetime of PCS. In literature, typically 10 years are assumed.^42^ In this study, we use the slightly reduced value of 8 years to account for assets being decommissioned before the end of life as well as hardware failure.

The OpEx estimations in this paper are based on the experiences from the project ALigN in which the authors participated.^43^ Note that our OpEx estimations are quite optimistic, as they do not contain replacement parts in case of component failures. This is in line with the slightly pessimistic lifetime outlined in the previous paragraph.

The estimated real interest rate of 4% is based on a 6% project internal rate of return and a 2% inflation rate. The internal rate of return is in line with renewable energy plants that typically yield between 4% for solar PV and up to 10% for specialized technologies.^44^ The 2% inflation rate was chosen as it is the stated target inflation rate of the European Central Bank^45^ and because actual inflation as close to 2% historically in Germany.^46^ The current inflation rate is significantly higher as a result of the war between Russia and Ukraine, but did not affect the period studied in this paper.

### Data cleaning

#### Limiting time window

As the analysis relies on knowing the durations of charge events, it was necessary to discard all data from the last status change of PCSs onwards as the duration of the last status would be undefined.

#### Removal of PCSs with little status information

To generate useful time series data, EVSEs were only considered if they experienced at least two status changes with the valid time frame being between the first and the last status change. This was done to prevent unrealistically long timespans if PCSs stop reporting data.

#### Removal of PCSs from outside Germany

Some of the PCSs obtained in the original dataset were just outside of the German borders. We removed all PCSs that were not located within the boundaries of Germany as defined by the Federal Agency for Cartography and Geodesy (“Bundesamt für Kartographie und Geodäsie”).^47^

#### Removal of CDRs with unrealistic values

Unrealistic CDRs were removed from the dataset if their consumed energy was either equal to 0 or above 1 MWh. They were also removed if information about their rated power was unavailable or invalid. The valid ranges were selected as shown in Table 3.

**Table 3:** Valid combinations of connector types and power and voltage ranges

Connector	Valid rated power	Valid voltage
Type 2	10≤P≤43kW	U≥220V
Schuko	P≤4kW	U≥220V
CCS, CHAdeMO	P≥50kW	U≥400V

For Type 2 and Schuko, we converted values into phase-to-neutral values.

Overall, the filtering leads to a reduction in the number of PCSs in the sample from 27,800 to 22,200. Note that filtering in step (2) might filter out stations that were valid data points, but were simply never used. Unfortunately, it is not possible to differentiate PCSs that no longer exist or are inaccessible from those that exist, but were not used in the 3 years observed. However, we expect the latter group to be negligibly small.

### Data time series generation

The data provided by the industry partners contain only status changes and not an entire time series. The events therefore had to be translated into time series data. This was done in the following steps for each EVSE in a slightly different way depending on the calculated value (see also Table 4 for an example):

• Arrivals: If an event was started in a time step, the time step was marked as 1, otherwise 0. If periods with an unknown status occurred, a 1 would only be assigned if the status after the unknown period was different from the status before.
• Occupation: An EVSE was marked as 1 for all moments in time where the previous status was “occupied” and as 0 for all moments in time when the last reported status was “available.”
• Duration: Similar to “Starts,” but the duration in hours instead of a 1 was used in the time step when the event started. For all duration plots, the durations shown consequently correspond to when events were started.

**Table 4:** Example of how the status changes for an EVSE translates into the time series assuming an hourly resolution

Timestep [h]	1	2	3	4	5	6	7	8	9	10	11	12	13	14	15	16	17	18	19	20	21	22	23	24
Raw data		O			A		U		A					O				A		O		U	O	A
Starts []	0	1	0	0	0	0	0	0	0	0	0	0	0	1	0	0	0	0	0	1	0	0	0	0
Duration [h]		3												4						4				
Occupation []	0	1	1	1	0	0	0	0	0	0	0	0	0	1	1	1	1	0	0	1	1	1	1	0

“O” – EVSE became occupied, “A” – EVSE became available, and “U” that the status of the EVSE became unknown.

#### Methods for profitability estimation

This section outlines the methods used to estimate PCS profitability. This is done in a four-step process where each step is explained in further detail in the following subsections:

• Calculate the energy sold annually
• Estimate annual revenues using a set profit margin
• Annualize costs
• Compare costs and revenues

##### Energy sold annually

**Figure 5:**
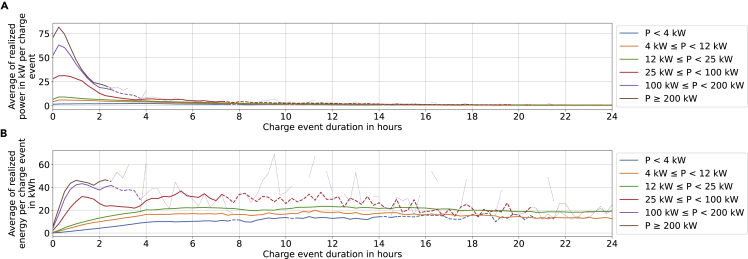
Average actual power flow and energy consumed for the charge events recorded in the CDR data (A and B) Average actual power flow (A) and energy consumed (B) for the charge events recorded in the CDR data. Note that the lower graph can be generated by multiplying the upper graph with the *x*-axis value. Example on how to read the graphs: for charge events lasting around 30 min (with quarter-hourly rounding windows) at chargers with a power rating above 200 kW, the actual power consumption was 57 kW when averaging over the entire event duration. Dashed and dotted lines indicate a lower data quality as outlined in section II of the supplementary information.

To estimate profitability, it is essential to quantify the units sold by an entity. In the case of EVSEs, the sold product is electricity. Although we do have information about the amount of energy sold for some PCSs for which the full list of CDRs is available, this is only the case for a minority of PCSs. For the vast majority of PCSs, we only see the energy transferred for a share of the events. For the PCSs where no full history of energy sales is available, an estimation method is required: CDRs allow us to calculate a relationship E(t) between the duration of a charging event and the energy consumed during such an event as shown in Figure 5. For charging events for which we have no information about the energy sales, but only a number of events and duration of events, they are estimated from the duration of the charging process.

**Figure 6:**
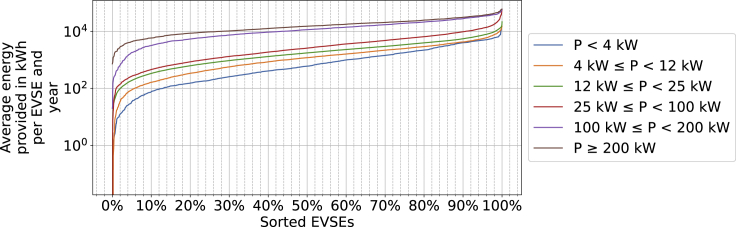
Average energy sold per EVSE categorized by power level. Example how to read: 10% of PCSs with a rated power between 12 and 25 kW sell 336 kWh or more per year

Once an amount of energy sold has been assigned to each charge event, the annual energy sold can simply be calculated by dividing the total energy sales by a specific EVSE by the duration that the EVSE was visible in our dataset measured in years. The result of this calculation corresponds to Figure 6. The approach can be summarized in the following equation:

$$E_{i,annual}=\frac{\sum_{j=0}^{n_{i}}E\left(t_{j}\right)}{T_{visible,i}}$$

where $E_{i,annual}$ is the annual energy sales of PCS i, $n_{i}$ is the total number of events at PCS i, and $T_{visible,i}$ is the duration for which the status of PCS i is known.

##### Annual revenues

The annual revenues are estimated for each EVSE by multiplying the annual energy sold by the assumed margin for the power level of the EVSE. The calculation can be summarized as follows.

$$R_{i,annual}=E_{i,annual}\cdot \lambda \left(P_{i}\right)$$

where $R_{i,annual}$ is the annual revenue of PCS i and λ(P) is the function returning sales margin per EVSE power. The sales margin is defined as revenue generated minus direct costs for service delivery, which, in our case, are the electricity costs of CPOs.

We chose to use sales margin instead of considering electricity sales and purchase prices. The reason for doing so is that both values vary widely across the industry, are trade secrets of the involved companies, and no centralized register exists that would allow a deduction of both values. Moreover, charge point operators (CPOs) and e-mobility service providers (EMPs) are frequently different actors. This means that the price paid for charging at the same PCS varies depending on the customer’s EMP. In this ecosystem, CPOs typically charge EMPs a fixed value for the usage of a PCS, which is comprised of the purchase cost of electricity and a margin to cover fixed costs. The margins used in this paper were checked with industry partners and considered realistic and are in line with typical end-customer ad-hoc prices shown in Figure 4.

##### Annualized costs

Capital expenses (CapEx) are singular payments whereas operational expenses (OpEx) and revenues are continuous cash flows. To allow comparison between the two, the CapEx are annualized using the annuity factor calculation below. Let ANF be the annuity factor, i the real interest rate defined as the nominal interest rate minus the inflation rate, n the number of years, and $CapEx_{Ann}$ the annualized CapEx.

$$ANF=\frac{{\left(1+i\right)}^{n}\cdot i}{{\left(1+i\right)}^{n}-1}CapEx_{Ann}=ANF\cdot CapEx$$

To determine the amount of energy that a PCS would need to sell in order to cover its costs, the following steps were taken:

• $CapEx_{Ann}$ was added to OpEx to calculate the full annual recurring costs RC:

$$RC=CapEx_{Ann}+OpEx$$

• The amount of energy required is found by dividing RC by the sales margin per unit of energy λ(P):

$$E_{required}=\frac{RC}{\lambda \left(P\right)}$$

##### Comparison

There are two ways of comparing costs and revenues. To determine whether an EVSE is able to refinance its costs or not, it is sufficient to compare $E_{required}$ with $E_{i,annual}$ and check whether sufficient energy has been sold. If $E_{i,annual}$ exceeds $E_{required}$, the EVSE will have sold enough electricity to cover its costs, otherwise not.

In the supplemental information, another view is presented: by calculating $R_{i,annual}-RC$, the net earnings or losses per EVSE can be calculated.

## Results

Results are split into two major sections. The first part called “Data visualisations” shows insights that can be obtained directly from aggregating and visualizing the data. The subsequent section “Profitability” provides estimates on the share of PCSs that are profitable.

### Data visualizations

This section provides aggregated plots of the data to outline key characteristics of PCS usage. The data are visualized in four categories, namely energy consumption per charge event, number of arrivals at stations, occupation levels, and duration.

Each section of the data visualization is comprised of the most relevant visualizations as well as a bullet point list of key aspects that should be included in all types of models that readers may create.

#### Energy consumption

Figure 5 shows the average power and energy consumption of charging processes split by rated power. Various aspects become apparent immediately:

• A high-rated power capacity of EVSEs correlates with high consumed energy.

Figures 5A and 5B show that as the rated power increases, the average energy consumed also increases. We find this to be accurate for all power levels up until 200 kW and charging durations up to 8 h (noise in the data increases afterward). CHAdeMO PCSs achieve a lower energy consumption than CCS PCSs (not shown).

The difference between the two connectors could be because of CHAdeMO only being installed on less than 4% of new vehicles (Own calculation based on the Federal Motor Transport Authority (“Kraftfahrtbundesamt”)^48^ and ADAC.^49^). CHAdeMO vehicles are consequently older on average and have a lower maximum acceptance rate (as this has gone up with time). The fact that no difference can be observed for chargers rated above and below 200 kW can be attributed to the fact that vehicles mostly cannot accept charge rates above ∼150 kW.

• Rated energy capacity remains largely unused.

Across all connector types and rated powers, the average energy consumed is between 30 and 60% of what the rated power would allow – even for the charging processes with a very short duration (see Figure 5). The median value shown in Figures S1C and S1E in the supplementary information at 10 kWh after 2 h correspond to ∼25 and ∼50% of the rated capacity for chargers rated at 12–25 kW and 4–12 kW, respectively.

For long sessions, this may be a result of battery filling. For short sessions, vehicle charging power may be insufficient, e.g. because of insufficient DC capacity or not using all phases in AC charging.^50^ Note that instantaneous power may still reach rated EVSE power. Unfortunately, our data only provide energy consumed and not instantaneous power.

• Start-up processes reduce consumed energy.

Charge events of 15 min show lower power consumption compared with slightly longer charge events as is evident in Figure 5A.

The charging processes recorded in our database are typically started upon authentication (i.e. holding the charging card to the terminal or using apps or websites). Afterward, the cable needs to be plugged in, power needs to be negotiated between vehicle and PCS, and the battery brought to a temperature range needs to accept the planned power flow. These steps possibly explain the low average power consumption.

• After 4–5 h, the energy consumed does not increase at AC chargers.

After some time, the energy consumed at three-phase AC chargers is capped. On average, this occurs after 4 h resulting in energy consumed of 16.3–20.3 kWh (see orange and green lines in Figure 5B, respectively). Figure S1 in the supplementary information complements this finding as it can be seen that after 5 h, 90% of charge events see no further increase in energy consumed for the power levels between 4 and 25 kW.

• Fast-chargers with more than 100 kW rated power recharge 40 kWh in ∼1 h, on average.

Typical current batteries for long-range cars have an energy capacity between 60 and 100 kWh.^51^ The average energy consumed reaches ∼40 kWh as shown in Figure 5B for EVSEs with more than 100-kW-rated power.

We can assume that this corresponds to a full recharge if some buffer is accounted for. The median values shown in Figure S1 in the supplementary information are similar.

**Figure 7:**
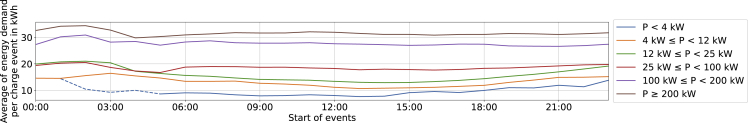
Energy demand per charge event by time of arrival at the PCS Example how to read: during charge events starting at 3 p.m. at EVSEs with a rated power between 12 and 25 kW, 12.9 kWh is consumed, on average. Dashed and dotted lines indicate a lower data quality, as outlined in section II of the supplementary information.

Instead of aggregating by event duration, it is also possible to aggregate charge events by starting time (see Figure 7). The figure shows that the energy consumed during AC charge events varies with the time of day. Charge events at AC PCSs create a high energy demand if started either in the evening or morning, as the blue, orange, and green lines in Figure 7A show. A similar pattern can be observed in the right column of Figure S1 in the supplementary in the top three plots. As will be outlined in section “duration,” this can be explained by the fact that events are significantly longer in the night or if started in the morning.

The observations presented all refer to averages across all EVSEs. The highly inhomogeneous pattern in Figure 6 means that 20% of EVSEs with a power rating between 4 and 100 kW sell approximately 55% of the energy in that group. For high-power DC chargers, this value is reduced to 40%.

#### Arrivals

**Figure 8:**
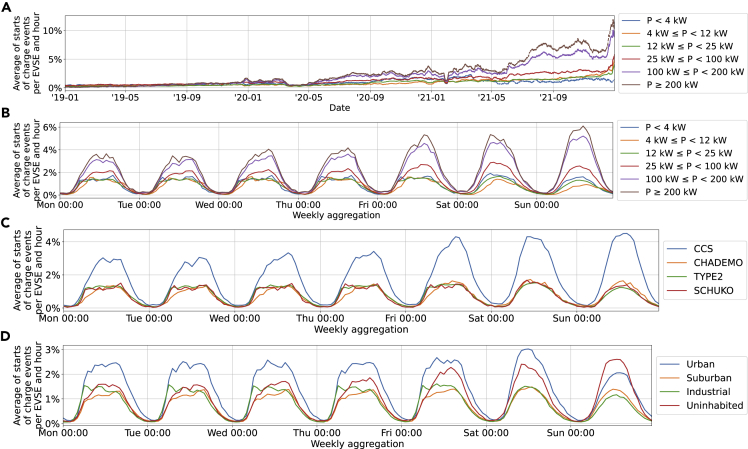
Number of charge events by starting time of events split by power level (A–D) Number of charge events by starting time of events split by power level over the observed period (A), aggregated over a week (B), split by connector type and aggregated over a week (C), and split by area type aggregated over a week (D). Example how to read: on an average Monday, at 3.0% of PCSs with a CCS connector a charge event starts between 2:30 and 3:30 p.m. (i.e. the hour around 3 p.m.). Additional aggregation levels as well as the same results for end of charge events are not shown here for the sake of conciseness, but are available in the appended dataset. Values in (A) are smoothed with a moving average of 1 week.

Figure 8 shows the arrivals of vehicles at PCSs split by the selection criteria employed in this paper. Some key observations are as follows:

• Number of arrivals per PCS has increased significantly over time.

Although the number of charge events increased for all PCS types over the observed period, the increase is strongest for EVSEs rated >100 kW (Figure 8A).

• Changes in mobility patterns because of the COVID-19 pandemic correlate with PCS usage.

The COVID-19 pandemic resulted in several lockdowns. The strongest reduction in mobility during the first lockdown (22 March – 4 May 2020) is reflected in decreasing charge events. Similar, but less dramatic effects are visible for the subsequent lockdowns in late 2020 and during the first half of 2021. The lower drops in usage correlate with a lower drop in mobility.^52^

• The number of arrivals rises with increasing EVSE-rated power.

PCSs with a higher rated power experience more charge events than those with a low-rated power (Figure 8B).

This is can be linked to the lower average charging duration for every single EVSE.

• CCS is used significantly more than CHAdeMO.

CHAdeMO connectors experience only about half as many charge events as CCS Figure 8C).

A possible explanation is that CCS is the standard for fast-charging in Europe used by virtually all European and US-American vehicles (including Tesla). EVSEs are however frequently equipped with both connectors,^2^ meaning that there are many more connectors per vehicle for CHAdeMO.

• Fast-charging is popular from Friday to Sunday, whereas AC charging is reduced on Sundays.

Fast-chargers experience on average more charge events on weekends as shown by the purple and brown line in Figure 8B. For Type 2, the effect is the opposite with lower usage on Sundays.

For fast-chargers, this is presumably related to people taking longer trips on weekends than on weekdays where commuting is more common.^53^ For Type 2 chargers, the lower number of events is likely the result of people going neither to work nor other leisure activities such as shopping.

• The amount of arrivals follows a bell-shape with small deviations for CCS and Type 2.

Most arrivals are almost symmetrical around noon with a slight increase between 6 and 9 a.m. for Type 2 connectors (Figure 8C), particularly in industrial areas (Figure 8D). For CCS, the bell-shape is slightly dented at midday with the two peaks at around 9–10 a.m. and 3–5 p.m. during workdays.

This is an indication of people charging their cars while commuting. Events at industrial Type 2 connectors are frequently started in the morning. Weekend usage is characterized by the lack of a dent for fast-chargers (Figure 8C).

When working with the data visualizations in Figure 8, it is important to keep the correlations shown in Table S4 in the supplementary information in mind, particularly for area types. Uninhabited areas are predominantly host to fast-chargers, whereas AC chargers are more frequent in urban and suburban settings. The plots are consequently not independent of each other.

#### Occupation

**Figure 9:**
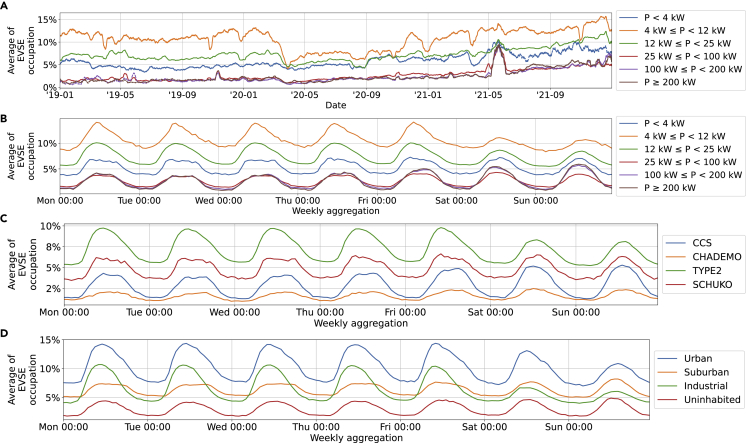
Share of PCSs occupied split by power level For a Figure360 author presentation of this figure, see https://doi.org/10.1016/j.isci.2022.105634. (A–D) Share of PCSs occupied split by power level over the observed period (A), aggregated over a week (B), split by connector type and aggregated over a week (C), and split by area type aggregated over a week (D). Example how to read: on an average Monday at 3 p.m., 9.85% of PCSs with a power rating between 12 and 25 kW were occupied. Values in (A) are a one-week moving average. Dashed and dotted lines indicate a lower data quality, as outlined in section II of the supplementary information.

Figure 9 shows the share of vehicles plugged in. Several trends are observable:

• Occupation is lower for fast-chargers.

Fast-chargers occupied less time than their slower counterparts (Figures 9A and B).

This might be surprising, given the fact that Figure 8 showed many more arrivals and departures for EVSEs with higher power ratings. The explanation for this phenomenon is that fast-charge events are much shorter than slow-charge events and therefore can service more vehicles while, simultaneously, also being much more available.

• Occupation is higher during the day.

The highest PCS occupation rate occurs during the day with the ratio between the highest and lowest occupation rate being 1.4–1.8 across all power levels (Figure 9B).

• Weekday-weekend patterns, CCS vs CHAdeMO, and long-running trends are similar to what can be observed for arrivals and departures.

The occupation follows the trends already visible in the number of arrivals and departures. The detailed explanations are consequently not repeated here (see section “arrivals”).

• The SD is large.

The SD (appended dataset, not shown) of all lines in Figure 9 is larger than the average. Typical values are between 20 and 40%.

This observation indicates a large diversity and randomness in the underlying dataset. As discussed in Figure 9, this was to be expected given the low predictive value of the power level, connector type, and surrounding area type alone.^34^ Individual circumstances must therefore be kept in mind when planning and operating a PCS.

**Figure 10:**
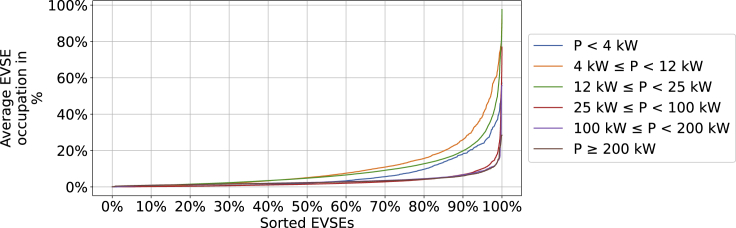
Average occupation per EVSE categorized by the power level

Figure 10 augments the findings by showing the difference in occupation between the individual EVSEs. Similar to the findings for energy sales, it can be concluded that there is a large variation among the PCS and their EVSEs.

#### Duration

**Figure 11:**
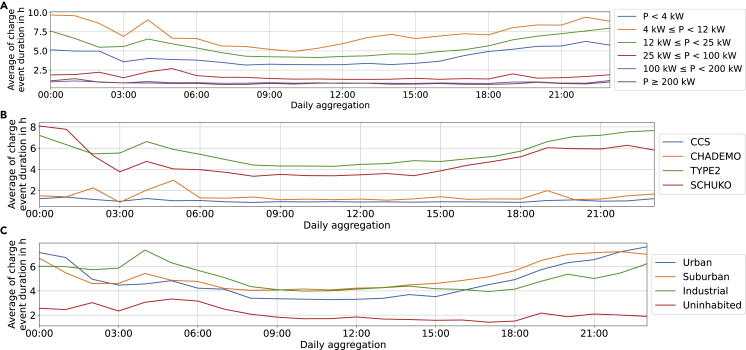
Duration of charge events by the time they started (A–C) Plot of the duration of charge events by the time that they started, aggregated by week, and split by rated EVSE power (A), connector type (B), and land usage (C).

Figure 11 shows the duration of the charging processes. The end of a charge event is defined as the moment when the vehicle is unplugged. The key findings are:

• AC charge events are longer if they start in the morning or evening than at noon.

Charge events at AC PCSs are longer if starting between 7 p.m. and 9 a.m. (see Type 2 and Schuko in Figure 6B). The effect is stronger for median values (appended data, not shown) where the median duration at noon is 2 h for all AC-chargers compared with 5.6 and 8.8 h at midnight for connectors rated 12–25 kW and 4–11 kW, respectively.

This is likely the combination of people parking their vehicles during either the night or workday.

• Events at industrial sites are on average especially long if started between 3 and 9 AM.

As Figure 11C shows, charge events in industrial areas are long if they are started between 3 and 9 a.m. The pattern is inverted in urban settings, which experience long occupations during the night.

This can be linked to people leaving their vehicles parked for the workday in industrial areas for the whole day. This is not possible in urban areas where parking is typically limited to 2–4 h.^54^

### Profitability

Table 5 shows a sample calculation of required energy sales. Based on assumptions regarding sales margin, costs, interest rate, and lifetime, the share of EVSEs is calculated that cover their costs through energy sales. Typical sales margin in the authors’ industry experience is marked in yellow. Cells marked in orange include revenue achievable from the greenhouse gas emission quota trading system (“THG-Quote”) in which PCS operators can receive funds from a levy imposed on petrol fuel sales. All these values were crosschecked with industry and academia experts and are in line with sales prices shown in Figure 4 as well as findings shown in the section “cost information.” Note that we do not include any change in consumer behavior in response to price changes in our analysis and assume a fixed PCS utilization independent of prices. An overview of how profitable individual EVSEs is rather than just the share of profitable PCSs can be found in section VI in the supplementary information.

**Table 5:** Estimation of share of profitable EVSEs given the assumptions made in the first five rows in the table with OpEx and interest rate being quite optimistic

		*P* < 4 kW	4 ≤ *P* < 12 kW	12 ≤ *P* < 25 kW	25 ≤ *P* < 100 kW	100 ≤ *P* < 200 kW	*P* ≥ 200 kW
Investment cost		2,000 €	6,000 €	9,000 €	50,000 €	100,000 €	200,000 €
Lifetime in years		8	8	8	8	8	8
Real interest rate		4%	4%	4%	4%	4%	4%
OpEx in €/a		122.2	76.3	77.2	136.4	128.8	117.6
Number of EVSEs per PCS		3.94	2.41	2.44	3.31	3.12	2.84

**PCS EVSEs independent of area type**							

Share profitable by sales margin	5 €ct/kWh	21.29%	1.77%	1.04%	0.08%	0.08%	0.11%
	10 €ct/kWh	**37.72%**	**11.63%**	**9.09%**	1.04%	0.42%	0.11%
	15 €ct/kWh	47.23%	23.15%	20.88%	3.07%	6.23%	0.11%
	20 €ct/kWh	*52.83%*	*33.04%*	*31.12%*	6.93%	14.99%	0.47%
	30 €ct/kWh	63.12%	46.51%	46.62%	**15.57%**	33.13%	6.26%
	40 €ct/kWh	68.99%	55.39%	56.81%	*24.46%*	47.77%	17.53%
	50 €ct/kWh	73.50%	61.40%	64.05%	31.60%	**58.92%**	**29.10%**
	60 €ct/kWh	77.06%	66.47%	69.45%	38.20%	*67.34%*	*40.79%*

**PCS EVSEs in an urban environment**							

Share profitable by sales margin	5 €ct/kWh	50.02%	7.73%	3.87%	0.39%	2.94%	No data
	10 €ct/kWh	**66.77%**	**34.55%**	**24.72%**	3.11%	2.94%	
	15 €ct/kWh	75.29%	48.92%	42.67%	10.59%	4.17%	
	20 €ct/kWh	*76.29%*	*58.43%*	*54.84%*	**19.11%**	11.92%	
	30 €ct/kWh	80.74%	67.76%	69.16%	*35.24%*	15.88%	
	40 €ct/kWh	84.31%	73.54%	76.67%	44.32%	34.80%	
	50 €ct/kWh	84.79%	78.64%	81.73%	53.71%	**53.07%**	
	60 €ct/kWh	85.88%	80.62%	85.11%	56.76%	*60.18%*	

**PCS EVSEs in a suburban environment**							

Share profitable by sales margin	5 €ct/kWh	14.79%	1.14%	0.56%	0.20%	0.40%	1.21%
	10 €ct/kWh	**31.18%**	**12.19%**	**7.07%**	0.65%	0.40%	1.21%
	15 €ct/kWh	40.54%	24.84%	18.55%	1.86%	2.20%	1.21%
	20 €ct/kWh	*47.48%*	*36.38%*	*29.04%*	5.06%	5.23%	1.21%
	30 €ct/kWh	59.49%	50.47%	45.13%	**12.57%**	17.95%	6.06%
	40 €ct/kWh	66.75%	60.95%	55.77%	*21.45%*	28.77%	10.55%
	50 €ct/kWh	71.76%	65.75%	63.19%	27.57%	**37.31%**	**21.11%**
	60 €ct/kWh	76.06%	70.83%	68.86%	34.27%	*45.95%*	*31.37%*

**PCS EVSEs in an industrial environment**							

Share profitable by sales margin	5 €ct/kWh	19.74%	0.74%	0.69%	0.11%	0.25%	0.40%
	10 €ct/kWh	**37.26%**	**5.79%**	**6.08%**	1.43%	0.67%	0.40%
	15 €ct/kWh	48.64%	16.75%	16.89%	4.06%	7.26%	0.40%
	20 €ct/kWh	*54.96%*	*26.34%*	*26.84%*	8.70%	18.25%	1.22%
	30 €ct/kWh	64.97%	41.51%	43.23%	**18.79%**	33.36%	7.70%
	40 €ct/kWh	68.24%	49.93%	54.02%	*28.08%*	45.62%	22.22%
	50 €ct/kWh	73.46%	56.83%	61.59%	36.52%	**55.74%**	**36.31%**
	60 €ct/kWh	75.95%	62.41%	67.18%	43.94%	*63.71%*	*45.47%*

**PCS EVSEs in an uninhabited environment**							

Share profitable by sales margin	5 €ct/kWh	5.00%	0.73%	0.16%	0.08%	0.17%	0.18%
	10 €ct/kWh	**12.85%**	**1.36%**	**2.50%**	0.62%	0.58%	0.18%
	15 €ct/kWh	16.78%	4.78%	7.02%	1.53%	7.67%	0.18%
	20 €ct/kWh	*18.45%*	*8.07%*	*12.06%*	3.95%	17.42%	0.34%
	30 €ct/kWh	20.63%	16.35%	22.21%	**10.17%**	40.91%	6.06%
	40 €ct/kWh	24.75%	24.95%	32.31%	*18.47%*	58.57%	17.06%
	50 €ct/kWh	40.21%	34.26%	41.71%	24.41%	**71.04%**	**27.65%**
	60 €ct/kWh	51.59%	41.89%	48.34%	30.56%	*79.96%*	*40.94%*

The chosen values were crosschecked with industry partners and academia for their correctness. The calculation results are shown for sales margins ranging from 5 to 60 €ct/kWh as outlined in the rows in each block. Cells with bold font are the most realistic sales margins according to the authors’ experience and are in line with the sales prices shown in Figure 4. Cells with italic font include typical extra revenues from the greenhouse gas quota trading system where operators receive funds from a levy imposed on fossil fuel sales. The first block of results is the aggregate of all area types and the subsequent blocks are split by area type. Note that costs are constant across different area types to ensure comparability, which may conflict with reality.

PCSs with a high-power rating appear the most profitable with 16 (25 ≤ *P* < 100 kW) to 59% (100 ≤ *P* < 200 kW) of EVSEs achieving break-even by the sale of electricity. For slower chargers, the situation is more problematic. Although investment costs are a magnitude lower, electricity sales are also equally lower. These PCSs compete with household prices of ∼30 €ct/kWh while purchasing electricity at ∼20 €ct/kWh.^55^

The results show that the required margins are significantly higher than for petrol sales. Across all power levels, sales margins of several dozen cents are required to recover expenses from energy sales alone for the majority of PCSs.

This value is significantly higher compared with conventional gas stations that earned 10.97 €cents per liter Diesel and 11.84 per liter Eurosuper^56^ on a km base. A car consuming 7 L Diesel per 100 km^57^ would consequently generate a 77 €cents margin. If the same absolute margin were applied to a comparable vehicle consuming 20 kWh per 100 km, only 3.84 €cents margin per kWh remain. Using the stated consumption values, fuel prices of currently ∼2€ per liter lead to 14 € per 100 km are comparable with costs for highway charging where the market leader Ionity currently charges 79 €cents per kWh leading to 15.80 € per 100 km. AC chargers with typical costs of 30–40 €cents per kWh in turn are much cheaper.

## Discussion

The results and visualizations shown in this paper provide detailed insights into the way that PCSs in Germany are used. The value of the work lies in the ability to quantify behaviors and effects that previously had an anecdotal character. An example of this type of result is that it was suspected that AC PCSs are frequently used for parking without much energy flowing. Section “energy consumption” quantifies this by outlining how AC chargers are used far below their rated power, whereas DC chargers typically supply enough energy per session to recharge a typical EV. Other observations of similar nature are possible using the provided material.

The profitability estimation yields sensible results. For the profitability estimate, strong assumptions had to be made regarding investment costs for PCSs and readers may disagree with the values chosen. For this reason, the graphs in section VI in the supplementary information are provided. Readers may assume higher costs, which corresponds to moving the zero-line in the provided plots, or readers may assume different profit margins. This can be represented by rescaling the *y*-axis.

The empirical mathematical modeling in the supplemental information provides simple-to-use models when using the full dataset is impractical or not necessary. With an $R^{2}$-score of 90% or more across almost all ways of grouping stations, the models can be concluded to be reasonably accurate. The data size has successfully been reduced from 100’s of GBs to 6 or 12 numbers for each model.

Charge point operators, public planners, mobility experts, scholars in the fields of mobility and energy, and other groups benefit from the obtained results. Applications of the found values include estimating the profitability of future PCS sites, comparing the performance of the current portfolio with national averages, comparing modeling results with real-world data, understanding customer behavior, and many others.

Some limitations must be considered. For instance, Figure 8A shows the usage patterns of PCSs are far from constant over time. Reasons are among others the effect of the COVID-19 pandemic on human mobility as well as the exponential increase in the EV stock. When working with the average weekly usage data, readers should consequently ensure that the data used fit their assumptions regarding human mobility. As there have been such a large variety of situations, we decided to not display each possible combination of market updates and COVID-19 counter-measures. Instead, we invite scholars, who want to work with the data, to use the non-aggregated data together with the number of observations in the attached dataset. Alternatively, readers may alter the amplitude and offset values of the empirical models to their needs.

When combining the data visualizations and results of this paper, many conclusions can be drawn and only a short list is provided here for the sake of conciseness.

• Most PCSs are not profitable from the energy that they sell according to our estimation shown in Table 5. This is particularly true for the slower AC chargers (38%, 12%, and 9% for PCS in the power ranges <4 kW, 4–12 kW, and 12–25 kW, respectively). The lack of profitability is assumed to be a product of the lower margins compared with DC PCSs. This is comparable with gas stations’ small margins on gas sales.^58^^,^^59^ PCSs should therefore be combined with the more profitable in-store sales and other secondary services.
• There are strong differences between the PCSs in each category where the best-performing fast-charging EVSE with a power rating above 100 kW is estimated to deliver 61 MWh per year and the worst performing one is estimated to deliver only about 20 kWh.
• Clear day-night and weekday-weekend patterns can be seen. AC chargers are used predominantly during the week and less on Saturdays. Dynamic pricing and other incentive mechanisms should be implemented at PCSs to make sure that the low occupation of PCS during the night and other off-peak periods is avoided. In the project BeNutz LaSA, prediction algorithms^34^ and incentive strategies are created to achieve this goal.
• It is possible to represent PCS usage using a simple set of parameters fitted to the empirical data. These fits constitute mathematical models that provide both the mean values over the course of a week as well as the uncertainties in the same resolution. Most models are able to capture the dynamics to a high degree and reach $R^{2}$-values of 0.9 or above. The models are not able to capture specifics such as a slight dent for fast-charger use during midday or increased use in the morning for industrial areas. Constructing more complex models is possible, but the added complexity is too great compared with the gained accuracy in our opinion.
• PCSs are built with a higher power rating than what is currently used. For DC-charging, this can be explained by the fact that operators install power ratings that are sufficiently high to keep up with the rising DC power of vehicles in Germany.^12^ The same can, however, not be said about AC charging power which has been 11 kW for the vast majority of vehicles also in recent years. Installations with connectors rated at 22 kW would therefore be able to supply more vehicles virtually without loss of comfort using the same grid connection capacity.

### Limitations of the study

As already hinted at in the discussion, this study is subject to a number of limitations that we would like to address in this chapter. Limitations are provided in the same order as the results, namely first regarding the data visualizations and second regarding the profitability analysis.

The EV market and the charging infrastructure market are both highly dynamic with varying growth rates over the past years. This necessarily means that usage patterns and trends are subject to significant changes, whereas the trends that we describe in the accompanying text in the “data visualizations” subsections do describe stable trends, which are not fundamentally changing over the analyzed period, this is not necessarily true for numerical values, particularly if trends are projected into the future. The trends shown in this paper can therefore serve as a starting point for researchers and decision makers, but should be verified with current trends, particularly if this document is read quite some time after publication.

A second issue is plotting only the averages. We outline in the supplemental information why we chose to do so. A key issue arising from this choice is that a faulty impression might be created for distributions that are highly non-normal, such as the occupation duration. To tackle this problem, other types of visualizations are provided in the published dataset^60^ and we strongly encourage readers to also consult data in that dataset if a specific result or value is of critical importance. For details on this, please refer to the Data and code availability statement.

A limitation related to the previous one described is the fact that the values shown might convey a sense of regularity and uniformity of usage across the PCSs in Germany. As discussed in our previous publication,^34^ the patterns are highly station-specific and far from regular. This means that if the unit of observation is a single PCS, relying on the shown trends alone may blind one to rare events with a strong impact. An example of this is the fact that the average power drawn at fast chargers, as shown in Figure 5A barely exceeds 75 kW, but this does not mean that some charge events are not drawing the full power at some point during the charging process.

Regarding the profitability analysis, the key limitation is the fact that the share of profitable PCSs strongly depends on the assumed margins. Sales price, electricity purchase price, operating costs, installation costs, etc. are all highly site- and operator-dependent. Choosing a constant value across all PCSs in Germany is clearly a strong simplification – one that we believe to be necessary for the sake of interpretability, but a limitation nonetheless. If the reader disagrees with estimated values regarding costs or margins, Table 5 and the material in the supplemental information provide sufficient material to determine the share of profitable PCSs with one’s own assumptions. It is therefore important to understand that although our estimation of profitability margins is accurate and reasonable in our view, it is not the only legitimate estimate and readers may choose a different value.

## STAR★Methods

### Key resources table

**Table:** 

REAGENT or RESOURCE	SOURCE	IDENTIFIER
**Deposited data**		

Analyzed data	^60^	Mendeley Data: https://doi.org/10.17632/ddv53zsf9m.1

**Software and algorithms**		

Python version 3.8.10	^62^	www.python.org
Pandas version 1.4.1	^63^	www.pandas.pydata.org
Numpy version 1.22.3	^64^	www.numpy.org
Matplotlib version 3.5.2	^65^	www.matplotlib.org
Seaborn version 0.11.2	^66^	www.seaborn.pydata.org

### Resource availability

#### Lead contact

Further information and requests for resources should be directed to and will be fulfilled by the lead contact, Christopher Hecht (Christopher.Hecht@isea.rwth-aachen.de). Please also include batteries@isea.rwth-aachen.de in all correspondence.

#### Materials availability

As this paper is a pure data analysis, no physical material was used.

### Method details

The methods used to create the shown results are outlined in the main body of the document. This section is consequently limited to a brief overview of the methodology.

For the generation of time series and aggregation plots in the first results section, the datasets supplied by industry were translated into time series data and aggregated using the aggregation functions available in the pandas^63^ and numpy^64^ library. The aggregations were subsequently analyzed for relevant trends and patterns. The plots shown and the accompanying text represent the most relevant and insightful findings that could be identified.

For the profitability analysis, the recharged energy per EVSE and year had to be calculated since this information was not available for all PCSs. This was done by looking up the average consumed energy for an event of the given duration and power level from the data shown in Figure 5. Once the annual electricity sale could be estimated, the annual margin per EVSE could be calculated by multiplying the sold energy with the assumed margin per kWh. This value was then compared to the investment and operating costs per EVSE to determine which stations were profitable and which ones not.

Computations were performed using the tools indicated in the key resources table on a custom server equipped with 30 AMD EPYC 7542 CPUs and a memory of 1.15 TB running Ubuntu 20.04.

**Table:** Legend and column titles for supplemental tables

**1 Chapter**		

The chapter in the paper to which the data shown belong		

**2 Description**		

A short description of the displayed type of data		

**3 Time aggregation**		

1	Not a time series	The data are not a time series and therefore cannot be aggregated
2	Daily	The data are aggregated on a daily level with an hourly resolution
3	Weekly	The data are aggregated on a weekly level with an hourly resolution
4	No aggregation	The data were not aggregated across time, although it is a time series

**4 Filter**		

1	Rated power	Charging station connectors are grouped by their rated power The grouped power levels are “P < 4kW,” “4kW ≤ P < 12 kW,” "12kW ≤ P < 25kW,” “25kW ≤ P < 100kW,” “100kW ≤ P < 200kW,” and “P ≥ 200kW”
2	Outlet type	Charging station connectors are grouped by their outlet type. The outlet types are “CCS,” “CHAdeMO,” “Type 2,” and “Schuko”
3	Area type	Charging station connectors are grouped by the area they are located in. The area types are “Urban,” “Suburban,” “Industrial,” and “Uninhabited”

**5 Metric**		

1	Average	The average of the quantity given in the description and aggregated by time and filter as outlined in the previous two sections is calculated
2	SD	The SD around the average of the quantity given in the description and aggregated by time and filter as outlined in the previous two sections is calculated
3	Median	Same as the average, but median instead of average values were calculated
4	Number of data points	The number of data points available to calculate average, median, and SD for each point in the plot is given. This value should be used if own aggregations are made by the reader to ensure that any derived average is correctly weighted by the number of datapoints in the original dataset
5	Deciles	The data are shown in 10% steps with the 50%-line corresponding to the median value. The 0%-line shows the line corresponding to the lowest data points and the 100%-line shows the line corresponding to the highest data points
6	EVSE distribution	The average value per EVSE is calculated and all EVSEs are ordered from a low to high value. Individual EVSE data points are not shown, but rather the 0.01%-steps in the data
7	Feature correlation matrix	The cross-correlation in this category is shown

**6 Unit**		

1	kWh	Kilowatthours
2	#	Number of datapoints in the underlying dataset
3	%	Percent
4	pp	Percentage points (i.e. the absolute difference between percentage values)
5	h	Hours

**7 Single category**		

For decile plots, only one element in the set used for filtering is shown per table. If the column “Single category” contains, for instance, “P < 4kW,” then the decile plot is the one for all stations with less than 4 kW of power.		

**8 Link to datasheet**		

A clickable link to the datasheet in which the data are contained

**Glossary**

**Table:** 

Term	Definition
Rated power/energy	The maximum power/energy that a system is able to provide Synonyms: installed, available
Realized power/energy	The actual power/energy that flew during a charge event
CCS	Acronym for Combined Charging System. A fast-charging connector standard
CHAdeMO	Acronym for CHArge de MOve. A fast-charging connector standard
Type 2	The three-phases connector standard used in Europe
Schuko	Single-phase plug and socket standard that is also used for normal household applications
Fast-charging	Refers to charging with high power. Traditionally, power ratings of 43 kW and more were considered. This paper defines fast-charging starting at 100 kW, given the otherwise long charging durations.
Charge event	The chain of events starting with a user authenticating at a PCS and ending with the unplugging of the vehicle
AC	Acronym for Alternating Current
DC	Acronym for Direct Current
EVSE	Acronym for Electric Vehicle Supply Equipment The device powering one or many connectors out of which only one can be used at a time (e.g. if CHAdeMO and CCS are installed at the same EVSE and only one can be used)
Connector	The physical connection point on the vehicle or PCS. The shape is defined by the connector standard.
PCS	Acronym for Charging Station A collection of EVSEs
CDR	Acronym for Charge Detail Record A data entry containing details about a charge event such as the start and end time, energy transferred, and EVSE used

## Data Availability

• Numerical data and visualisations have been deposited in our Mendeley data repository^60^ and are publicly available as of the date of publication. The data is structured in parallel to the categories “Energy consumption,” “Arrivals,” “Occupation,” “Duration,” and “Ends.” The first four correspond to the “data visualizations” subsections and the latter is structurally similar to “arrivals”, but was not shown due to the low additional amount of information gained if arrivals and duration are known. Data and visualisations are aggregated on a daily or weekly level or not at all. The available file formats are either csv for the raw data or png and svg for image files. When using any of the attached material, please cite this paper. • The raw utilization data cannot be shared, as this is proprietary property of the industry partners. • This paper does not report original code as the analysis is based on proprietary data that cannot be shared. All pre-processing steps have been described in sections “data sources and pre-processing” and “methods for profitability estimation”. • Any additional information required to reanalyze the data reported in this paper is available from the lead contact upon request.

## Supplemental information

Document S1. Figures S1, S3, S6–S8, and S22 and Tables S2, S4, S5, and S9–S21

Figure360. An Author Presentation of Figure 9

## References

[1] CAIT Climate Data Explorer via. Climate Watch (2020). Emissions by sector. https://ourworldindata.org/emissions-by-sector#annual-co2-emissions-by-sector.

[2] Bundesnetzagentur (2022). Ladesäulenkarte. https://www.bundesnetzagentur.de/DE/Sachgebiete/ElektrizitaetundGas/Unternehmen_Institutionen/HandelundVertrieb/Ladesaeulenkarte/Ladesaeulenkarte_node.html.

[3] Almaghrebi A., Shom S., Al Juheshi F., James K., Alahmad M. (2019). 2019 IEEE Transportation Electrification Conference and Expo (ITEC).

[4] Almaghrebi A., Juheshi F.A., Nekl J., James K., Alahmad M. (2020). 2020 IEEE Transportation Electrification Conference & Expo (ITEC).

[5] Almaghrebi A., Aljuheshi F., Rafaie M., James K., Alahmad M. (2020). Data-driven charging demand prediction at public charging stations using supervised machine learning regression methods. Energies.

[6] van den Hoed R., Helmus J.R., Vries R. de, Bardok D. (2013). 2013 World Electric Vehicle Symposium and Exhibition (EVS27).

[7] Wolbertus R., van den Hoed R., Maase S. (2016). Benchmarking charging infrastructure utilization. WEVJ.

[8] Wolbertus R., Kroesen M., van den Hoed R., Chorus C. (2018). Fully charged: an empirical study into the factors that influence connection times at EV-charging stations. Energy Pol..

[9] Gerritsma M.K., AlSkaif T.A., Fidder H.A., Sark W.G.v. (2019). Flexibility of electric vehicle demand: analysis of measured charging data and simulation for the future. WEVJ.

[10] van der Kam M., van Sark W., Alkemade F. (2020). Multiple roads ahead: how charging behavior can guide charging infrastructure roll-out policy. Transport. Res. Transport Environ..

[11] Zhang Z., Chen Z., Xing Q., Ji Z., Zhang T. (2022). Evaluation of the multi-dimensional growth potential of China's public charging facilities for electric vehicles through 2030. Util. Pol..

[12] Figgener J., Hecht C., Haberschusz D., Bors J., Spreuer K.G., Kairies K.-P., Stenzel P., Sauer D.U. (2022).

[13] (2022). Bundesministerium für Digitales und Verkehr, NOW GmbH and Nationale Leitstelle Ladeinfrastruktur. https://www.standorttool.de/strom/deutschlandnetz/.

[14] Shell Recharge Solutions (2022).

[15] Wolff S., Madlener R. (2020). Willing to pay? Spatial heterogeneity of e-vehicle charging preferences in Germany. SSRN J..

[16] UScale (2021). Elektromobiltätsstudie zur öffentlichen Ladeinfrastruktur 2021. https://uscale.digital/emobility-studie-zur-oeffentlichen-ladeinfrastruktur-2021/.

[17] Betz J., Walther L., Lienkamp M. (2017). 2017 IEEE Intelligent Vehicles Symposium (IV).

[18] Efthymiou D., Chrysostomou K., Morfoulaki M., Aifantopoulou G. (2017). Electric vehicles charging infrastructure location: a genetic algorithm approach. Eur. Transp. Res. Rev..

[19] Ji D., Zhao Y., Dong X., Zhao M., Yang L., Lv M., Chen G., Bi Y., Chen G., Deng Q., Wang Y. (2018). Embedded Systems Technology.

[20] Cocca M., Giordano D., Mellia M., Vassio L. (2018). 2018 21st International Conference on Intelligent Transportation Systems (ITSC).

[21] Pahlavanhoseini A., Sepasian M.S. (2019). Scenario-based planning of fast charging stations considering network reconfiguration using cooperative coevolutionary approach. J. Energy Storage.

[22] Bryden T.S., Hilton G., Cruden A., Holton T. (2018). Electric vehicle fast charging station usage and power requirements. Energy.

[23] Baresch M., Moser S. (2019). Allocation of e-car charging: assessing the utilization of charging infrastructures by location. Transport. Res. Pol. Pract..

[24] Erbaş M., Kabak M., Özceylan E., Çetinkaya C. (2018). Optimal siting of electric vehicle charging stations: a GIS-based fuzzy Multi-Criteria Decision Analysis. Energy.

[25] Figgener J., Tepe B., Rücker F., Schoeneberger I., Hecht C., Jossen A., Sauer D.U. (2021).

[26] Tepe B., Figgener J., Englberger S., Sauer D.U., Jossen A., Hesse H. (2022). Optimal pool composition of commercial electric vehicles in V2G fleet operation of various electricity markets. Appl. Energy.

[27] Fischer M., Hardt C., Michalk W., Bogenberger K. (2022). Charging or Idling: Method for Quantifying the Charging and the Idle Time of Public Charging Stations. https://www.researchgate.net/publication/357900426_Charging_or_Idling_Method_for_Quantifying_the_Charging_and_the_Idle_Time_of_Public_Charging_Stations.

[28] Calearo L., Marinelli M., Ziras C. (2021). A review of data sources for electric vehicle integration studies. Renew. Sustain. Energy Rev..

[29] Hecht C., Das S., Bussar C., Sauer D.U. (2020). Representative, empirical, real-world charging station usage characteristics and data in Germany. eTransportation.

[30] Mortimer B.J., Bach A.D., Hecht C., Sauer D.U., Doncker R.W. de (2021). Public charging infrastructure in Germany — a utilization and profitability analysis. J. Mod. Power Syst. Clean Energy.

[31] Mortimer B.J., Hecht C., Goldbeck R., Sauer D.U., De Doncker R.W. (2022). Electric vehicle public charging infrastructure planning using real-world charging data. WEVJ.

[32] Wolbertus R., van den Hoed R. (2017). Charging station hogging: A data-driven analysis.

[33] EEA (2018).

[34] Hecht C., Figgener J., Sauer D.U. (2021). Predicting electric vehicle charging station availability using ensemble machine learning. Energies.

[35] Hecht C. (2020). BeNutz LaSA. https://benutzlasa.de/.

[36] Alberer L. (2020).

[37] Auf der Maur A., Brüggeshemke N., Kutschera M. (2020).

[38] Plank-Wiedenbeck U., Harder R., Kohl P. (2021).

[39] Langer A. (2018).

[40] emobilitaet.business Redaktion (2020). Öffentliche Ladestationen: alle Infos auf einen Blick. https://emobilitaet.business/wissensdatenbank/ladeinfrastruktur/6973-oeffentliche-ladestationen.

[41] (2019). WD 5: Wirtschaft und Verkehr, Ernährung, Landwirtschaft und Verbraucherschutz.

[42] Waxmann N., Heckmann J., Pyschny H. (2021).

[43] Stippich A. (2022). Project ALigN. https://www.isea.rwth-aachen.de/cms/ISEA/Forschung/Projekte/Oeffentliche-Projekte/Laufende-Projekte/%7Esrwf/Projekt-ALigN-Steckbrief/?lidx=1.

[44] van Nuffel L., Hoogland O. (2020).

[45] European Central Bank (2022). Monetary policy. https://www.ecb.europa.eu/ecb/tasks/monpol/html/index.en.html.

[46] Destatis (2022). Result 61111-0001. https://www-genesis.destatis.de/genesis/online?operation=previous&levelindex=2&step=2&titel=Result&levelid=1660719107265&acceptscookies=false#abreadcrumb.

[47] (2019). Bundesamt für Kartographie und Geodäsie. https://gdz.bkg.bund.de/index.php/default/open-data/verwaltungsgebiete-1-250-000-ebenen-stand-01-01-vg250-ebenen-01-01.html.

[48] Kraftfahrt-Bundesamt (2022). Bestand am 1. Januar 2021 nach Zulassungsbezirken und Gemeinden. https://www.kba.de/DE/Statistik/Fahrzeuge/Bestand/ZulassungsbezirkeGemeinden/zulassungsbezirke_node.html.

[49] ADAC (2020). Automarken & Modelle. https://www.adac.de/rund-ums-fahrzeug/autokatalog/marken-modelle/.

[50] Hecht C., Jan F., Sauer D.U. (2021).

[51] Electric Vehicle Database (2022). Useable battery capacity of full electric vehicles. https://ev-database.org/cheatsheet/useable-battery-capacity-electric-car.

[52] Statistisches Bundesamt Deutschland (2022). Experimentelle Daten - Mobilitätsindikatoren mit Mobilfunkdaten. https://www.destatis.de/DE/Service/EXDAT/Datensaetze/mobilitaetsindikatoren-mobilfunkdaten.html.

[53] Follmer R., Gruschwitz D. (2019).

[54] ADAC (2022). E-Ladesäulen: Parkbeschilderung of unklar. https://www.adac.de/rund-ums-fahrzeug/elektromobilitaet/laden/parken-e-ladesaeulen/.

[55] Federal grid agency, and federal Cartel office (2021).

[56] Gürsel M.B. (2020).

[57] DLR, DIW, KBA (2021).

[58] Crockett Z. (2021). Why most gas stations don’t make money from selling gas. https://thehustle.co/why-most-gas-stations-dont-make-money-from-selling-gas/.

[59] Horsley S. (2007). Gas Stations Profit from More Than Just Gas. https://www.npr.org/templates/story/story.php?storyId=10733468.

[60] Hecht C. (2022). Analysis of Electric Vehicle Charging Station Usage and Profitability in Germany based on Empirical Data.

[61] LichtBlick SE (2021). E-Mobilität: Ein Fall von Marktversagen. https://www.lichtblick.de/ladesaeulencheck21/.

[62] Python Software Foundation (2022). Python 3.8.10. https://www.python.org/downloads/release/python-3810/.

[63] The pandas development team (2022). pandas-dev/pandas: Pandas.

[64] Harris C.R., Millman K.J., van der Walt S.J., Gommers R., Virtanen P., Cournapeau D., Wieser E., Taylor J., Berg S., Smith N.J. (2020). Array programming with NumPy. Nature.

[65] Hunter J.D. (2007). Matplotlib: a 2D graphics environment. Comput. Sci. Eng..

[66] Waskom M. (2021). seaborn: statistical data visualization. J. Open Source Softw..

