# Supplementary material for: Analysis of electric vehicle charging station usage and profitability in Germany based on empirical data
Source: iScience. 2022 Nov 19;25(12):105634. doi: 10.1016/j.isci.2022.105634 (PMC9763839; doi:10.1016/j.isci.2022.105634)
Supplement: Document S1. Figures S1, S3, S6–S8, and S22 and Tables S2, S4, S5, and S9–S21 [file mmc1.pdf]

iScience, Volume *25*

Supplemental information

Analysis of electric vehicle charging station usage and profitability in Germany based on empirical data

Christopher Hecht, Jan Figgener, and Dirk Uwe Sauer

Supplementary information

I. Deciles of charge events by rated power (related to section "Energy consumption")

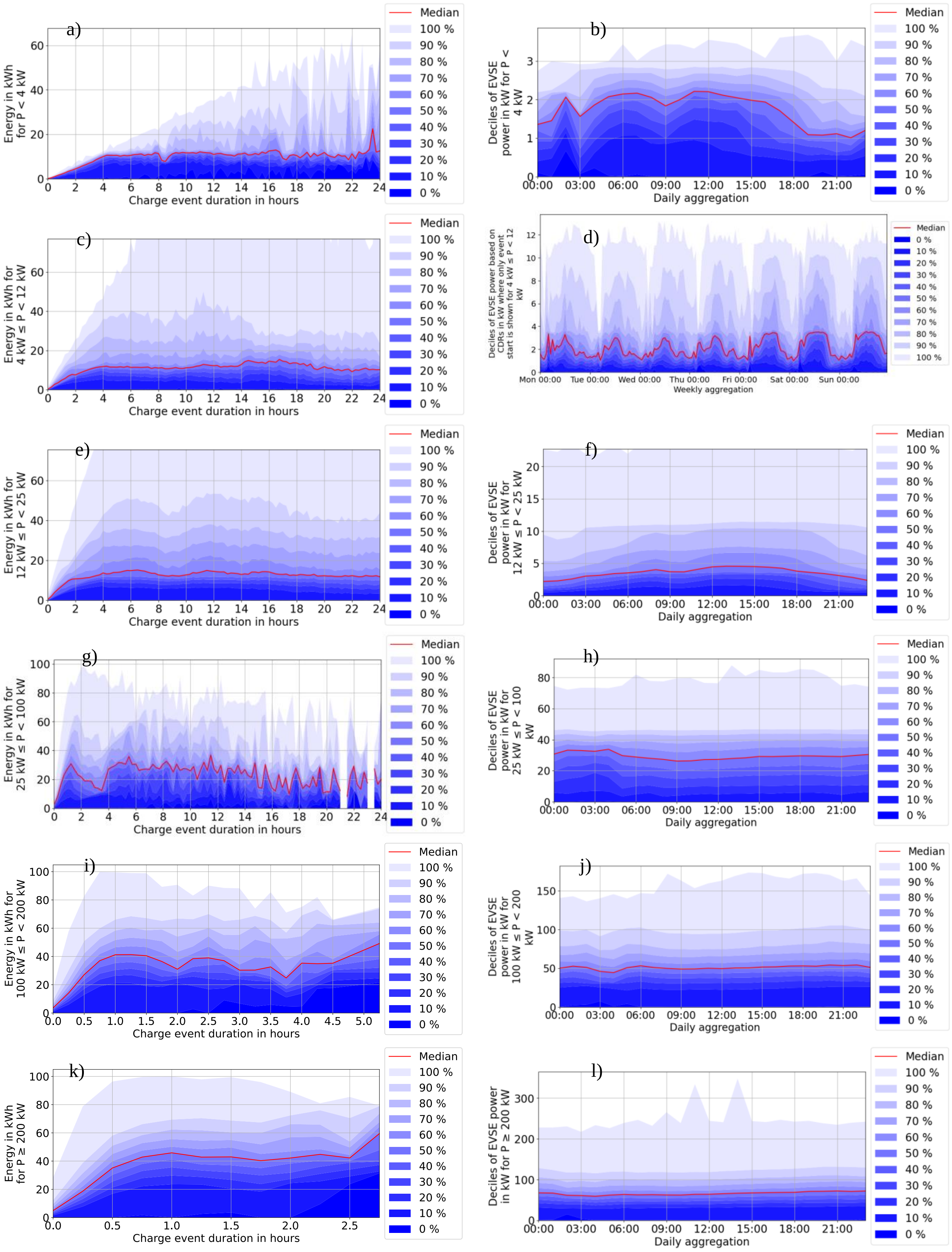

S1: Deciles of power flow for charge events recorded in the CDR data. The shade of blue indicates what percentage of events was below the fraction stated in the legend for each duration. The power ratings of the group of EVSEs, for which events are displayed, can be found on the y-axis of each plot with levels increasing in the standard order of this paper. For the daily aggregation shown on the right, the value plotted always corresponds to the power of events starting at the time and not over the entire event duration. Examples how to read: For charge events lasting around 15 minutes (with quarterhourly rounding windows) at EVSEs with a power rating above 200 kW, 80% of events were at a power level of less than ~73 kW when averaged over event duration (left plots). For charge events at EVSEs with a rated power between 12 and 25 kW and starting at midday, 50% experienced an average power of 4.45 kW over the entire event duration. Note that events lasting a time x do not influence the shown data for durations lower than x. Events lasting between 15 and 30 minutes consequently are not counted for the data point showing the power level of events lasting between 0 and 7.5 minutes. Note that no values are displayed if less than 10 events with the given duration occurred explaining the large white spaces particularly for fast-chargers. Related to Figure 5.

II. MEANING OF DIFFERENT LINE STYLES IN PLOTS (RELATED TO ALL LINE PLOTS IN THE MAIN DOCUMENT)

Line plots in this paper are either solid, striped, dotted or blank. The definition of these styles is given in the table below.

S2: Definition of line styles in all plots showing average values. Related to all line plots in the main document.

Line style	Meaning
Wide and solid	The data points forming the line consist of at least 100 observations each.
Medium width and dashed	The data points forming the line consist of at least 20 observations each.
Thin and dotted	The data points forming the line consist of less than 20 observations each.
Blank	No line is shown if no corresponding data points are available

Data points are generated in regular intervals across all plots these intervals generally are:

- every 15 minutes for plots showing a time window of 24 hours
- every 60 minutes for plots showing a weekly aggregation or no aggregation at all

III. CHARACERISATION OF PCS IN THE DATASET (RELATED TO SECTION "DATA SOURCES")

To gain an understanding of the PCS compromising the sample analysed in this paper, this section outlines several key statistics. S3 shows where PCSs are located according to the methodology outlined in section "PCS data" (main document). Nearly half of the PCSs are located in a suburban environment. All categories are, however, large enough that sufficient data points for a robust analysis are still available with above 2000 PCS located in uninhabited areas.

S4 expands the classification of PCS by showing the correlation between area type and the other classification categories power level and connector type. Relevant aspects to point out are the strong correlation of uninhabited areas with the fast-charging standards CCS and CHAdeMO. This is a result of many fast-chargers on highways being located in areas that we consider uninhabited. Reversely, most chargers in an urban or suburban setting are Type 2 Schuko with lower power levels.

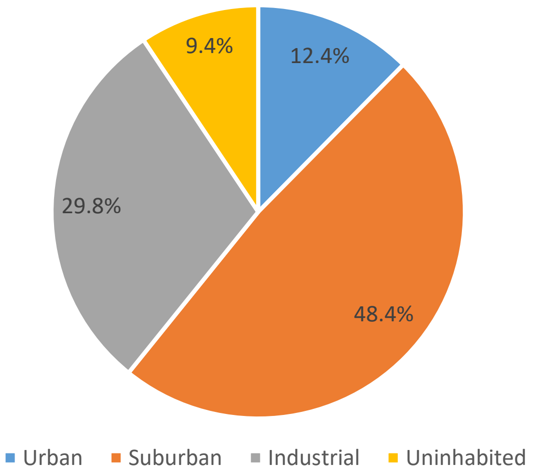

S3: Locations of PCS according to the categorization used in this paper. Related to section "Data sources".

S4: Correlation matrix of area types with the static properties power rating and connector type. Related to section "Data sources".

	Urban	Suburb.	Indus.	Uninhab.
P < 4 kW	0.007	0.060	-0.041	-0.041
4 kW ≤ P < 12 kW	0.009	-0.047	0.047	-0.031
12 kW ≤ P < 25 kW	0.052	0.097	-0.059	-0.130
25 kW ≤ P < 100 kW	-0.065	-0.125	0.082	0.153
100 kW ≤ P < 200 kW	-0.049	-0.093	0.004	0.199
P ≥ 200 kW	-0.045	-0.112	-0.012	0.248
CCS	-0.076	-0.165	0.040	0.290
CHAdeMO	-0.044	-0.075	0.030	0.128
Type 2	0.079	0.137	-0.027	-0.270
Schuko	0.007	0.057	-0.039	-0.040

To gain an understanding of what is near the PCS analysed in this study, we provide the distances to the nearest Points of Interest (POI) in OpenStreetMaps[1] in S5. The categorisation is identical to the one used in our previous publication[2]. To verify that the data used in this study is comparable to the PCSs registered in the governments registry, we provide the same values for those PCS as well. Both values typically only differ by a few percent with strong differences only showing up for POIs that are seldom (Aeroway and Marina/ Harbour). We consider this a satisfactory result.

S6 extends this view by showing how many PCSs have how many types of POIs nearby. 91% of PCSs have at least three types of POI categories within a 500 m radius, which indicates that the vast majority is constructed in areas with amenities to be used during the charging process.

Another way of looking at the data is to check how many other POI categories are nearby if a certain type of POI is near the PCS. Gas stations, aeroways, and shops score lowest in this category. Gas stations and shops can frequently be found with little other building structure nearby. This can be explained by the fact that both amenities are often located at central transport hubs or road intersections. Aeroways in turn are so large that there is little space for other amenities. Railway stations, car sharing sites, bars, pubs, clubs, cinemas, and hospitals in turn are POI categories where, if they are close to a PCS, many other POIs will also be nearby.

S5: Average and median distance of the PCSs in the dataset as well as the share of PCSs, which have at least one POI within a 500 m radius around them. Values in italic are for the register of the German government[3]. Related to section "Data sources".

	Average in km	Median in km	Share with POI within 500 m
Hotel/ Accommoda-tion	0.93	0.54	47.0%
	0.89	*0.54*	*47.2%*
Public Building	0.66	0.44	55.3%
	0.65	*0.45*	*54.9%*
College/ University/ School	0.64	0.33	65.9%
	0.64	*0.34*	*65.6%*
Bar/Pub/Club	1.37	0.64	42.9%
	1.33	*0.63*	*43.9%*
Restaurant	0.42	0.22	77.3%
	0.42	*0.22*	*77.0%*
Cafe/ Fast-food	0.66	0.26	69.3%
	0.63	*0.25*	*70.4%*
Hospital/ Healthcare	1.32	0.64	41.0%
	1.28	*0.63*	*41.5%*
Clinic	0.77	0.34	61.5%
	0.77	*0.34*	*61.0%*
Bank/Post	0.83	0.44	54.0%
	0.83	*0.47*	*52.2%*
Place of Worship	0.63	0.40	59.6%
	0.64	*0.42*	*57.6%*
Convenience	0.45	0.20	77.8%
	0.43	*0.19*	*77.8%*
Recreational Sites	0.90	0.46	53.7%
	0.88	*0.45*	*53.9%*
Shop	0.41	0.13	82.5%
	0.40	*0.13*	*81.9%*
Marketplace	6.18	3.61	11.3%
	5.99	*3.35*	*11.2%*
Sports facilities	0.75	0.50	49.9%
	0.75	*0.51*	*49.2%*
Tourism	0.82	0.49	50.5%
	0.81	*0.50*	*50.0%*
Cinema/ Museum	1.29	0.68	41.6%
	1.28	*0.68*	*41.1%*
Theme Park/ Stadium	3.42	2.41	5.8%
	3.33	*2.32*	*6.1%*
Aeroway	26.22	22.03	0.3%
	25.89	*21.59*	*0.4%*
Gas-Station	1.01	0.62	40.7%
	0.96	*0.60*	*41.5%*
Marina/ Harbour	13.26	9.12	2.3%
	12.60	*8.03*	*2.5%*
Car Rental/ Sharing	5.57	2.69	17.8%
	5.38	*2.45*	*18.2%*
Railway Station	2.68	1.53	17.5%
	2.57	*1.46*	*17.7%*

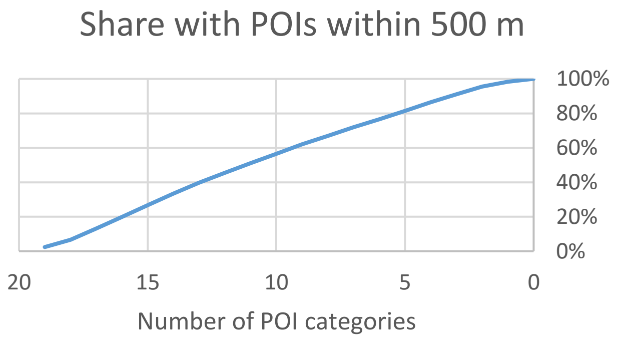

S6: Share of PCSs in the dataset by the number of POI categories that can be found within a 500 m radius. Related to section "Data sources".

IV. Statistical validity (Related to section "Data sources and pre-processing)

The values in this paper are representative in the sense that they are based on a large dataset comprising the majority of PCSs in the country. We, nevertheless,

made several choices about visualization of results and analysis which are justified in the following.

A. Choice of features

The major features that this paper analyses are power level, location of the PCS, and connector type. These features were chosen based on previous analysis[4, 5] where these showed consistently low p-values below 0.05 and comparatively high predictive power[6]. These two aspects combined indicate that there is a strong and non-random correlation between the chosen features and the displayed results.

Other features such as the weather, vacation periods, and long weekends are not considered. The reason for not including the weather is that neither temperature nor precipitation had strong predictive quality[6]. All three features are highly seasonal. Since the displayed data encompasses the COVID-19 pandemic, such a seasonality might be misleading since there were much stronger mobility restrictions in place during the lock-downs during the winters as compared to summers[7]. Any observed effect may therefore be correlated to the unusual circumstances caused by the pandemic and not necessarily be valid in the future anymore.

B. Average values

The results reported in this document are all presented in terms of average values since this comparatively simple measure can be applied across all reported results and is easily understood in the wider scientific community. The main problem with this approach is the fact that the data is typically not normally distributed around the average as S1 shows. This leads to the averages being substantially higher than the median.

Metrics such as plotting deciles (i.e. 10%-steps) or medians could arguably be used in some visualisations. For the sake of conciseness, we decided against showing these metrics in the paper. Instead, they are provided in the attached dataset for readers wishing to perform a deep-dive into the data.

C. Standard deviations and uncertainty

The usage patterns of PCSs are subject to a large number of random factors and consequently cannot be explained using a few simple features alone. This can be measured using the standard deviation around the average. As outlined in the previous section, the data is not normally distributed around the average, which would normally not allow for the usage of standard deviation. Since the metric is again one understood by the wider community, we nevertheless calculated it for all displayed plots and added it to the additional material. The second measure is the decile plot, which provides a more detailed insight in the data at the expense of requiring one plot per line a line-plot.

V. Empirical modelling (Related to sections "Data sources and pre-processing" and "Discussion")

In this section, an approach is presented with which the obtained results can be compressed into a set of short formulas. Goodness of fit indicators as well as fitted parameters are provided.

A. Methodology

The patterns found for energy consumption, arrivals, occupation share, and duration were translated into fitted empirical mathematical models. Each model is described by a formula providing the average value as well as a formula for the uncertainty estimate. The latter is necessary since a heteroscedasticity exists for most values. This approach allows generating sample data for an EVSE that is described by the same properties as were used for fitting a model such as power level or area type.

To simplify usage, we aim to create models with the fewest parameters possible that still accurately represent the shape of the empirical curves. Accuracy is measured using the share of variance explainable by the model (R^2) as defined below:

$$R^2 = 1 - \frac{\sum_i (y_i - f_i)^2}{\sum_i (y_i - \bar{y})^2}$$

where y_i is the real data at point i, f_i is the function value at i, and $\bar{y}$ is the mean across all y_i. Note that a weighted R^2 was used for all models except for the duration model, where the weight corresponds to the number of data points and can therefore be represented using the same formula keeping in mind that the same y may appear for many i.

Across all models, the goal is to achieve an R^2 of 90% meaning that the model is able to explain 90% of variance. We consider this a reasonable level for simple-to-use models. The models can be divided into cyclical formulations that were used to represent weekly arrival and occupation patterns and non-cyclical formulas to model energy consumption and duration. The approaches are outlined in the following sections. An example of the approach can be found in S7.

1) Cyclic model for arrivals and occupation

The data visualisations showed that there are strong daily cycles both for number of cars arriving as well as the share of occupied stations. These cycles are different on weekdays and weekends. The simplest mathematical function to capture such behaviour is the combination of two sine functions as defined below.

$$f(h) = \begin{cases} A_{wd} \cdot \sin\left(h \cdot \frac{2\pi}{24} - \theta_{wd}\right) + C_{wd} & for\ h \leq 5 \cdot 24 \\ A_{we} \cdot \sin\left(h \cdot \frac{2\pi}{24} - \theta_{we}\right) + C_{we} & otherwise \end{cases}$$

where A_{wd} and A_{we} are the amplitude factors on weekdays and weekends, respectively, ϕ_{wd} and ϕ_{we} are the phase shift factors on weekdays and weekends, respectively, C_{wd} and C_{we} are the offset factors on weekdays and weekends, respectively, h is the hour of the week in the interval [0,168[.

The described function can be used to describe both the mean value as well as the standard deviations. The full model describing the number of starts per charging station and hour as well as the occupation value can consequently be defined as follows:

.

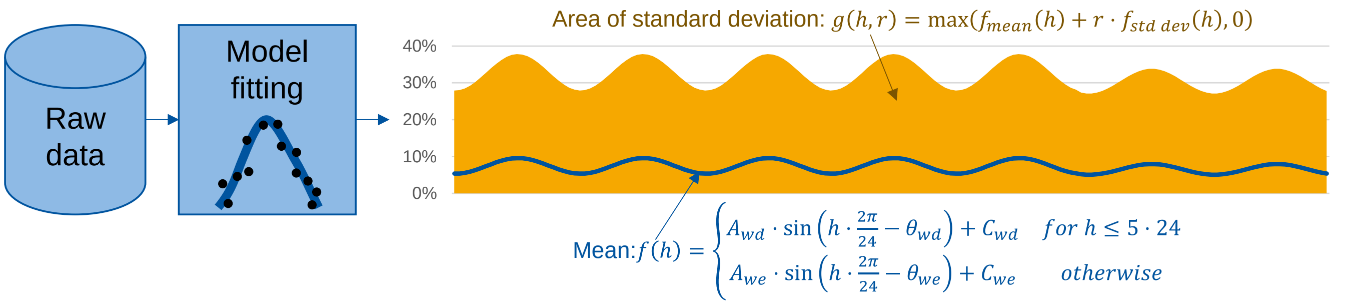

S7: Schematic of how raw data translates into empirical models through model fitting on the example of the occupation of Type 2 connectors as shown in Figure 9. Blue lines indicate mean values and the shaded area in yellow shows the standard deviation (which is large as noted earlier). Shown formulas correspond to those in section V.A.1). Related to Figure 9.

$$g(h,r) = \max(f_{mean}(h) + r \cdot f_{std\ dev}(h), 0)$$

where the first part of the summation describes the mean and the second part the standard deviation with r being a randomly chosen value from a unit normal distribution. The maximum function ensures that only non-negative values are calculated as negative occupation or number of starts is not sensible

2) *Non-cyclic model for duration and energy consumption*

While the data shows slight variations over the course of the day with longer charge events starting in the evening as compared to the rest of the day, the variation between charge events started at the same time is significantly greater. It was therefore decided to generate a mathematical model that is capable of capturing the random variation between the duration of charge events and not incorporate the intra-day variation. Capturing both would lead to an excessively complex model. If readers require the intra-day variation, we suggest creating a look-up table based model using the attached dataset. The model formulation suggested in this paper is given below:

$$t(x) = a \cdot e^{b \cdot (x-c)} with\ x \in [0,1]$$

where a, b, and c are fitted parameters, x is a random variable with equal probability across the interval [0,1], and t is the duration of the charge event.

Once the duration of a charge event has been defined, the next and last step is to estimate the amount of energy that was consumed during the event. The following formula has been chosen since the minimum function captures the fact that the amount of energy recharged will eventually stagnate even at very long durations due to the battery being full. The natural log function in turn is able to capture the fact that the amount of energy transferred is usually very high in the first phases of the charge event and gradually decreases.

$$m(t) = \min(i, j \cdot log_e(t + k))$$

Where i, j, and k are fitted parameters.

The function can be used to describe both the mean value as well as the standard deviation due to the inherent heteroscedasticity. The amount of energy transferred E during a charge event of duration t can consequently be described as follows. Similar to the cyclic models, the maximum function ensures non-negative values for energy transferred.

$$E(t,r) = \max(m_{mean}(t) + r \cdot m_{std\ dev}(t), 0)$$

3) *Combination of models*

The provided models may be chained to generate a realistic time series for a charging station usage. A pseudo-algorithm follows the following steps:

1. Set hour of the week $h = 0$.
2. Evaluate $g(h,r)$ parametrised for arrivals using randomly chosen r from the unit normal distribution and round the result to the nearest integer.
3. Add $g(h,r)$ vehicles to the waiting queue. For each vehicle, randomly choose x from the even distribution on the interval [0,1] and mark the arrival time as $h + x$.
4. If $h == 167$, terminate.
5. Set $h = h + 1$and return to step 2.

This algorithm will create a random time series containing the number of arriving vehicles over the course of the week. To calculate the energy that the EVSE needs to provide, the following pseudo-algorithm may be used:

1. Start at the first vehicle in the waiting queue.
2. Evaluate $t(x)$ using an randomly chosen x from the even distribution on the interval $[0,1]$.
3. Evaluate $E(t,r)$ using the found t and a randomly chosen r from a unit normal distribution.
4. Mark the station as occupied from h until $h + t$ with a total energy consumption E over the charge event.
5. If the current vehicle is the last vehicle in the queue, terminate.
6. If the next vehicle in the queue was added at a h where the EVSE is still occupied, move h to the first moment in time where the EVSE is available (i.e. the new car waits until the EVSE is free).
7. Return to step 2 for the next vehicle in the queue.

B. Results

S8 shows an example comparison when plotting the raw data and when plotting the empirical model outputs

fitted using the methodology described in section III. Visually, the curves are nearly identical indicating a good fit. S9 - S11 augment this observation by providing the R^2-value of the functions. As can be seen, almost all models achieve an R^2-value of 0.9 or higher. This proves that the chosen models are a reasonable choice and are able to capture the dynamics of the underlying dataset to a highly accurate degree. The models tend to perform weaker for the slowest charging events at stations rated below 4 kW, particularly for energy consumption. We consider this acceptable since these EVSEs play only a subordinate role in the overall infrastructure with 379 installations at the end of 2021. The patterns are further much more irregular than those of other charging levels, which makes it harder to capture the dynamics properly.

Certain features such as the slightly increased usage of AC chargers in the morning cannot be reproduced. Given the overall low impact of these effects, we consider this lack of precision acceptable as model complexity is significantly reduced.

We did not fit models for the energy consumption when classified based on connector or area type. The reason for that is that the power level is the most important factor to quantify the amount of energy per charging event.

The parameters of the fitted models are not shown here for the sake of conciseness, but can be found in the appendix III to allow fellow researchers to reconstruct mathematical models for their own purposes.

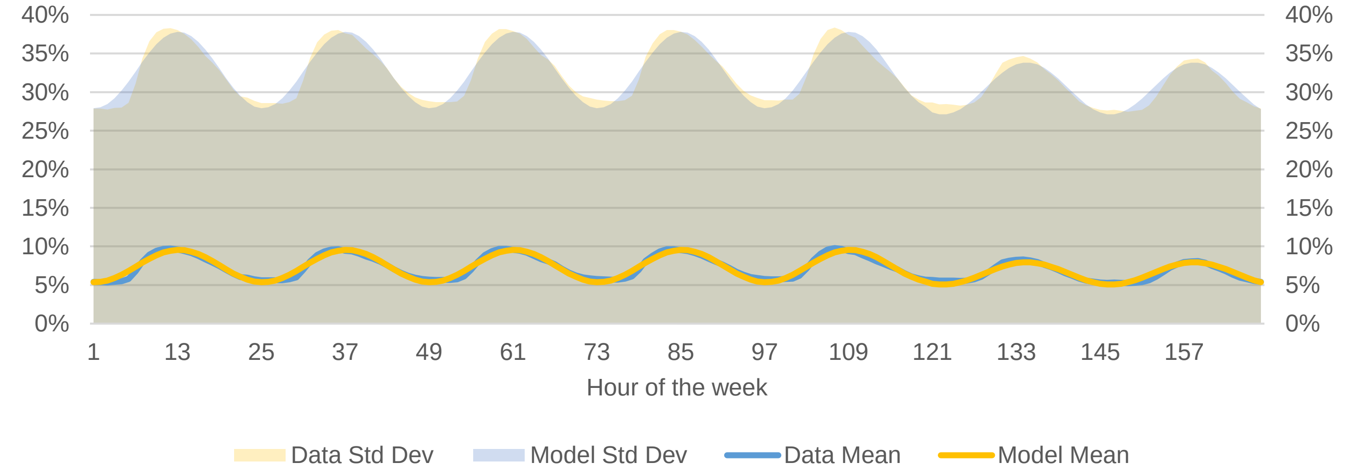

S8: Comparison of mean and standard deviation of data and of the fitted model for the occupation status of Type 2 connectors as shown in Figure 9 (main document). The fitted model is created with the formulas in section V.A.1) and the values shown for Type 2 in S14. Related to sections "Data sources and pre-processing" and "Discussion".

S9: R^2 of empirical models parametrized by power level. $\bar{x}$ indicates the R^2 for the model describing the mean and σ indicates the R^2 for the models describing the standard deviation. Related to sections "Data sources and pre-processing" and "Discussion".

		P < 4 kW	4 kW ≤ P < 12 kW	12 kW ≤ P < 25 kW	25 kW ≤ P < 100 kW	100 kW ≤ P < 200 kW	P ≥ 200 kW
Starts	$\bar{x}$	0.89	0.91	0.93	0.96	0.95	0.94
	σ	0.89	0.89	0.91	0.96	0.96	0.95
Duration		0.99	0.99	0.99	0.99	0.97	0.97
Energy cons.	$\bar{x}$	0.93	0.97	0.99	0.95	0.99	0.98
	σ	0.69	0.93	0.99	0.97	0.96	0.96
Occu-pation	$\bar{x}$	0.92	0.91	0.95	0.96	0.94	0.94
	σ	0.89	0.89	0.94	0.95	0.93	0.93

S10: R^2 of empirical models parametrized by area type. $\bar{x}$ indicates the R^2 for the model describing the mean and σ indicates the R^2 for the models describing the standard deviation. Related to sections "Data sources and pre-processing" and "Discussion".

		Urban	Suburban	Industrial	Uninhabited
Starts	$\bar{x}$	0.91	0.94	0.90	0.95
	σ	0.90	0.93	0.90	0.95
Duration		0.99	0.99	0.99	1.00
Occu-pation	$\bar{x}$	0.92	0.94	0.94	0.95
	σ	0.91	0.89	0.94	0.95

S11: R^2 of empirical models parametrized by connector standard. $\bar{x}$ indicates the R^2 for the model describing the mean and σ indicates the R^2 for the models describing the standard deviation. Related to sections "Data sources and pre-processing" and "Discussion".

		CCS	CHAdeMO	TYPE2	SCHUKO
Starts	$\bar{x}$	0.95	0.96	0.92	0.89
	σ	0.96	0.96	0.91	0.89
Duration		0.99	0.98	0.98	0.99
Occu-pation	$\bar{x}$	0.95	0.93	0.95	0.92
	σ	0.95	0.88	0.93	0.88

C. *Fitted values*

This section provides the fitted values for the mathematical models developed in this paper. Please refer to section III for a definition of shown variables and to section III for an overview of the goodness of fit for all fits.

1) *Energy consumption*

S12: Fitted values for energy consumption grouped by power level. $\bar{x}$ are the fitted values for the function providing the mean and σ are the fitted values for the function providing the standard deviation at each point. The row indices in the first column correspond to the variables shown in section V.A.2). Related to section "Energy consumption".

	P < 4 kW		4 kW ≤ P < 12 kW		12 kW ≤ P < 25 kW		25 kW ≤ P < 100 kW		100 kW ≤ P < 200 kW		P ≥ 200 kW	
	$\bar{x}$	σ	$\bar{x}$	σ	$\bar{x}$	σ	$\bar{x}$	σ	$\bar{x}$	σ	$\bar{x}$	σ
i	14.601	8.356	16.401	13.739	21.428	17.698	28.619	19.072	42.043	17.615	45.727	17.001
j	5.324	2.470	9.657	6.792	12.139	8.477	39.414	20.863	66.247	25.707	74.690	28.969
k	0.721	0.315	0.886	0.713	0.962	0.897	0.997	0.995	1.047	1.198	1.092	1.237

2) *Arrivals*

S13: Fitted values for occupation levels grouped by power level. $\bar{x}$ are the fitted values for the function providing the mean and σ are the fitted values for the function providing the standard deviation at each point. The row indices in the first column correspond to the variables shown in section V.A.1). Related to Figure 8 in section "Arrivals".

	P < 4 kW		4 kW ≤ P < 12 kW		12 kW ≤ P < 25 kW		25 kW ≤ P < 100 kW		100 kW ≤ P < 200 kW		P ≥ 200 kW	
	$\bar{x}$	σ	$\bar{x}$	σ	$\bar{x}$	σ	$\bar{x}$	σ	$\bar{x}$	σ	$\bar{x}$	σ
A_{wd}	0.0078	0.0502	0.0070	0.0465	0.0072	0.0452	0.0113	0.0618	0.0175	0.0766	0.0199	0.0830
θ_{wd}	1.6322	1.6554	1.9085	1.9529	1.6572	1.6996	1.8057	1.8168	1.8347	1.8246	1.8186	1.8130
C_{wd}	0.0089	0.0876	0.0085	0.0879	0.0087	0.0881	0.0117	0.1031	0.0181	0.1296	0.0206	0.1375
A_{we}	0.0085	0.0525	0.0053	0.0413	0.0071	0.0465	0.0134	0.0686	0.0248	0.0965	0.0281	0.1045
θ_{we}	1.7595	1.8199	2.1896	2.2202	1.8584	1.9038	1.8012	1.8366	1.7912	1.8109	1.7826	1.8015
C_{we}	0.0083	0.0837	0.0054	0.0680	0.0071	0.0787	0.0129	0.1081	0.0231	0.1445	0.0260	0.1523

S14: Fitted values for occupation levels grouped by connector type. $\bar{x}$ are the fitted values for the function providing the mean and σ are the fitted values for the function providing the standard deviation at each point. The row indices in the first column correspond to the variables shown in section V.A.1). Related to Figure 8 in section "Arrivals".

	CCS		CHAdeMO		Type 2		Schuko	
	$\bar{x}$	σ	$\bar{x}$	σ	$\bar{x}$	σ	$\bar{x}$	σ
A_{wd}	0.0167	0.0756	0.0067	0.0462	0.0068	0.0445	0.0068	0.0471
θ_{wd}	1.8278	1.8252	1.8026	1.8122	1.5925	1.6387	1.6099	1.6365
C_{wd}	0.0172	0.1253	0.0072	0.0829	0.0082	0.0856	0.0077	0.0811
A_{we}	0.0223	0.0912	0.0077	0.0501	0.0067	0.0453	0.0074	0.0497
θ_{we}	1.8028	1.8276	1.7879	1.8119	1.7857	1.8326	1.7382	1.8003
C_{we}	0.0209	0.1368	0.0079	0.0880	0.0067	0.0765	0.0071	0.0773

S15: Fitted values for occupation levels grouped by area type. $\bar{x}$ are the fitted values for the function providing the mean and σ are the fitted values for the function providing the standard deviation at each point. The row indices in the first column correspond to the variables shown in section V.A.1). Related to Figure 8 in section "Arrivals".

	Urban		Suburban		Industrial		Uninhabited	
	$\bar{x}$	σ	$\bar{x}$	σ	$\bar{x}$	σ	$\bar{x}$	σ
A_{wd}	0.0125	0.0603	0.0063	0.0426	0.0075	0.0490	0.0088	0.0543
θ_{wd}	1.6981	1.7423	1.7535	1.7653	1.3899	1.4750	1.7473	1.7568
C_{wd}	0.0148	0.1135	0.0075	0.0819	0.0086	0.0875	0.0094	0.0931
A_{we}	0.0123	0.0607	0.0070	0.0458	0.0065	0.0474	0.0125	0.0692
θ_{we}	1.8711	1.9246	1.7822	1.8238	1.7523	1.7965	1.7546	1.7870
C_{we}	0.0126	0.1037	0.0070	0.0785	0.0063	0.0750	0.0116	0.1020

3) *Occupation*

S16: Fitted values for occupation levels grouped by power level. $\bar{x}$ are the fitted values for the function providing the mean and σ are the fitted values for the function providing the standard deviation at each point. The row indices in the first column correspond to the variables shown in section V.A.1). Related to Figure 9 in section "Occupation".

	P < 4 kW		4 kW ≤ P < 12 kW		12 kW ≤ P < 25 kW		25 kW ≤ P < 100 kW		100 kW ≤ P < 200 kW		P ≥ 200 kW	
	$\bar{x}$	σ	$\bar{x}$	σ	$\bar{x}$	σ	$\bar{x}$	σ	$\bar{x}$	σ	$\bar{x}$	σ
A_{wd}	0.0158	0.0256	0.0212	0.0203	0.0210	0.0275	0.0121	0.0255	0.0158	0.0297	0.0172	0.0330
θ_{wd}	1.8107	1.8206	1.7360	1.6396	1.6725	1.6436	1.8730	1.8776	1.9105	1.9404	1.8806	1.8937
C_{wd}	0.0539	0.2167	0.1109	0.3052	0.0777	0.2587	0.0265	0.1454	0.0262	0.1317	0.0253	0.1237
A_{we}	0.0157	0.0250	0.0097	0.0080	0.0144	0.0186	0.0134	0.0268	0.0222	0.0390	0.0244	0.0433
θ_{we}	1.9108	1.9166	2.3159	2.3162	2.0437	2.0558	1.9473	1.9982	1.8800	1.9336	1.8692	1.9148
C_{we}	0.0518	0.2131	0.0967	0.2898	0.0680	0.2444	0.0267	0.1446	0.0304	0.1376	0.0303	0.1322

S17: Fitted values for occupation levels grouped by connector type. $\bar{x}$ are the fitted values for the function providing the mean and σ are the fitted values for the function providing the standard deviation at each point. The row indices in the first column correspond to the variables shown in section V.A.1). Related to Figure 9 in section "Occupation".

	CCS		CHAdeMO		Type 2		Schuko	
	$\bar{x}$	σ	$\bar{x}$	σ	$\bar{x}$	σ	$\bar{x}$	σ
A_{wd}	0.0158	0.0299	0.0053	0.0124	0.0210	0.0285	0.0135	0.0229
θ_{wd}	1.8870	1.8931	1.9443	2.0295	1.6255	1.6054	1.8128	1.8289
C_{wd}	0.0278	0.1400	0.0156	0.1125	0.0746	0.2540	0.0492	0.2083
A_{we}	0.0201	0.0359	0.0063	0.0142	0.0144	0.0193	0.0136	0.0226
θ_{we}	1.8963	1.9354	1.9914	2.1204	1.9608	1.9689	1.9046	1.9132
C_{we}	0.0301	0.1422	0.0167	0.1157	0.0651	0.2396	0.0475	0.2051

S18: Fitted values for occupation levels grouped by area type. $\bar{x}$ are the fitted values for the function providing the mean and σ are the fitted values for the function providing the standard deviation at each point. The row indices in the first column correspond to the variables shown in section V.A.1). Related to Figure 9 in section "Occupation".

	Urban		Suburban		Industrial		Uninhabited	
	$\bar{x}$	σ	$\bar{x}$	σ	$\bar{x}$	σ	$\bar{x}$	σ
A_{wd}	0.0334	0.0346	0.0115	0.0152	0.0318	0.0480	0.0132	0.0282
θ_{wd}	1.6905	1.6663	1.9543	1.9868	1.4182	1.4099	1.7521	1.7318
C_{wd}	0.1065	0.2953	0.0639	0.2370	0.0701	0.2438	0.0302	0.1587
A_{we}	0.0235	0.0241	0.0142	0.0194	0.0110	0.0165	0.0146	0.0284
θ_{we}	2.0605	2.0913	1.9747	1.9882	1.8485	1.8429	1.8609	1.8872
C_{we}	0.0923	0.2782	0.0625	0.2347	0.0509	0.2129	0.0300	0.1555

D. Duration

S19: Fitted values for duration grouped by power level. The row indices in the first column correspond to the variables shown in section V.A.2). Related to Figure 11 in section "Duration".

	P < 4 kW	4 kW ≤ P < 12 kW	12 kW ≤ P < 25 kW	25 kW ≤ P < 100 kW	100 kW ≤ P < 200 kW
a	0.5965	1.2419	0.9612	0.6886	1.1249
b	3.5853	3.9183	3.6815	3.2904	2.0675
c	0.1869	0.3051	0.2816	0.5903	0.9499

S20: Fitted values for duration grouped by connector type. The row indices in the first column correspond to the variables shown in section V.A.2). Related to Figure 11 in section "Duration".

	CCS	CHAdeMO	Type 2	Schuko
a	0.1089	2.0693	1.1645	1.3400
b	2.2934	2.7643	3.7904	3.6218
c	-0.1326	1.0572	0.3496	0.4094

S21: Fitted values for duration grouped by area type. The row indices in the first column correspond to the variables shown in section V.A.2). Related to Figure 11 in section "Duration".

	Urban	Suburban	Industrial	Uninhabited
a	1.3329	2.4024	0.4967	0.8078
b	3.6272	3.9389	4.2201	5.4705
c	0.3990	0.5619	0.2413	0.6614

VI. PROFITABILITY SPLIT BY STATIONS (RELATED TO SECTION "PROFITABILITY")

S22 shows an overview of the annualized cash flow using the assumptions given in Table 2 (main document) for the most probable profit margin. A positive cash flow corresponds to a CS EVSE making a profit whereas a negative cash flow means that annualized investment cost and operational costs exceed estimated revenue. Related to Table 5 in section "Profitabililty").

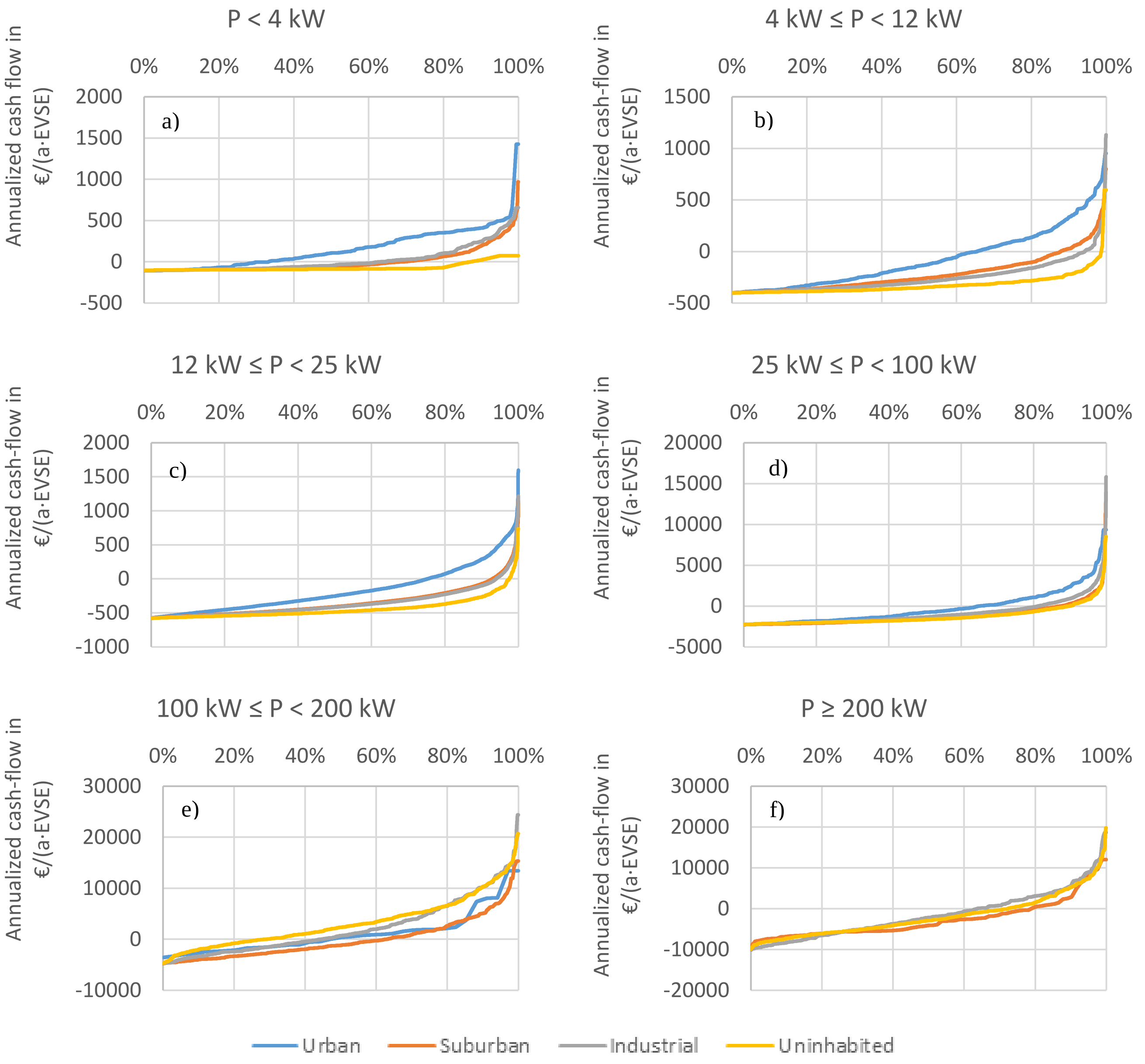

S22: Annualized cash flow in € per year and CS EVSE split by power level and location of the PCS. The annualized cash flow includes annualized investment costs and the margin earned from electricity sales, both as defined in Table 5 (main document). Revenues from GHG emission quota trading are not included. PCSs were sorted by annually sold electricity and evenly spread between 0% and 100%. Example how to read: 50% of EVSEs with a power rating between between 25 kW and 100 kW in an urban environment achieved a positive annualized cash flow of 358.39 € per year and EVSE. Related to Table 5 in section "Profitability".

VII. REFERENCES IN THE SUPPLEMENTARY MATERIAL

1. OpenStreetMap contributors (2017). Germany dump retrieved from https://planet.osm.org, https://www.openstreetmap.org/.
2. Mortimer, B.J., Hecht, C., Goldbeck, R., Sauer, D.U., and Doncker, R.W. de (2022). Electric Vehicle Public Charging Infrastructure Planning Using Real-World Charging Data. WEVJ *13,* 94.
3. Bundesnetzagentur (2022). Ladesäulenkarte, https://www.bundesnetzagentur.de/DE/Sachgebiete/ElektrizitaetundGas/Unternehmen_Institutionen/HandelundVertrieb/Ladesaeulenkarte/Ladesaeulenkarte_node.html.

4. Hecht, C., Das, S., Bussar, C., and Sauer, D.U. (2020). Representative, empirical, real-world charging station usage characteristics and data in Germany. eTransportation *6,* 100079.
5. Olk, C., Trunschke, M., Bussar, C. and Sauer, D.U., eds. (2019). Empirical Study of Electric Vehicle Charging Infrastructure Usage in Ireland (Xiamen, China: IEEE).
6. Hecht, C., Figgener, J., and Sauer, D.U. (2021). Predicting Electric Vehicle Charging Station Availability Using Ensemble Machine Learning. Energies *14,* 7834.
7. Statistisches Bundesamt Deutschland (2022). Experimentelle Daten - Mobilitätsindikatoren mit Mobilfunkdaten, https://www.destatis.de/DE/Service/EXDAT/Datensaetze/mobilitaetsindikatoren-mobilfunkdaten.html.
